# GENESIS-SHIELD: an interpretable ensemble for anomaly detection in CRISPR genomic-workflow security

**DOI:** 10.3389/frai.2026.1893433

**Published:** 2026-09-18

**Authors:** Prabakaran C., Kannadasan R.

**Affiliations:** School of Computer Science and Engineering, Vellore Institute of Technology, Vellore, Tamil Nadu, India

**Keywords:** attention mechanism, blockchain integrity, CRISPR anomaly detection, ensemble learning, ethics-aware systems, genomic security, interpretable AI, temporal anomaly detection

## Abstract

**Introduction:**

The rapid advancement of CRISPR-based gene editing has introduced digital-workflow security and integrity challenges: unauthorized modifications, temporal inconsistencies, and duplicated provenance records can compromise the auditability of editing logs. These are distinct from the biological risks of editing itself; our focus is the security of the *digital record*. Existing single-component detectors capture only one facet of these threats.

**Methods:**

We present GENESIS-SHIELD, a multi-component ensemble whose novelty lies in the *integration* of established techniques for CRISPR-workflow security rather than in any single new algorithm. It combines four components—a Hierarchical Blockchain Merkle Tree with Bloom filters and a train-registry integrity check, an adaptive cross-layer entropy/KL analyzer, an interpretable decision-tree ethics rule system, and a structural temporal-consistency detector—whose weights are set by Bayesian optimization of a recall-oriented (F_2_) validation objective. We benchmark against classic (Isolation Forest, One-Class SVM, LOF) and modern (XGBoost, autoencoder, and Deep-SVDD) baselines trained on identical features, and report a leave-one-component-out ablation, bootstrap confidence intervals, and isotonic calibration.

**Results:**

On a *synthetic* benchmark of 50,000 records (5% anomalies, eight categories), the ensemble attains AUC-ROC 0.982 (95% CI 0.973–0.990) and AUC-PR 0.841, with the proposed weighted detector reaching recall 0.978 at precision 0.500 (F_1_ 0.661). A supervised XGBoost on the same features is competitive-to-superior (AUC-PR 0.892, F_1_ 0.854), and the deep Attention-LSTM remains ineffective under class imbalance (F_1_ 0.096). Ablation shows the ethics and structural components carry the signal while the entropy component is redundant (removing it leaves metrics unchanged). Isotonic calibration reduces expected calibration error from 0.028 to 0.002.

**Discussion:**

These results are a proof-of-concept on rule-defined synthetic data; the high per-category detection reflects the benchmark's construction and does not establish real-world security. Validation on real editing logs, adversarial testing, and multi-objective coverage remain necessary before deployment.

## 1 Introduction

### 1.1 Background and motivation

The rapid advancement of CRISPR-Cas9 and related gene editing technologies has revolutionized biomedical research and therapeutic interventions, enabling precise modifications to genetic material with unprecedented accuracy (Anzalone et al., 2019; Porto et al., 2020). Clinical applications of gene editing have expanded dramatically, with multiple CRISPR-based therapies entering human trials for conditions ranging from sickle cell disease to cancer (Frangoul et al., 2021; Stadtmauer et al., 2020). However, this technological revolution has simultaneously introduced critical security, safety, and ethical challenges that demand immediate attention from the research community (Brokowski and Adli, 2019; Cwik, 2020).

The integrity and security of genomic editing data have emerged as paramount concerns in both research laboratories and clinical settings. Several threat vectors compromise the trustworthiness of gene editing workflows: unauthorized modifications to restricted genes, temporal anomalies indicating system breaches or data manipulation, extreme phenotypic outcomes signaling dangerous editing combinations, and cryptographic vulnerabilities in data verification systems (Yao et al., 2020; Li et al., 2021). The consequences of such security breaches extend beyond data corruption—they can result in irreversible harm to patients, compromise of research integrity, and erosion of public trust in gene therapy technologies (Cai et al., 2020; Ploug and Holm, 2020).

Traditional cybersecurity mechanisms designed for conventional data systems prove inadequate for genomic editing data due to several unique characteristics. First, genomic data exhibits high dimensionality with complex interdependencies between genes, making pattern-based anomaly detection challenging (Chen et al., 2020). Second, editing transactions possess strong temporal dependencies where the sequence and timing of modifications carry semantic meaning (Wang T. et al., 2020). Third, ethical constraints impose domain-specific rules that cannot be captured by generic security policies (Liao, 2019). Fourth, the irreversible nature of genetic modifications demands near-zero false negative rates, as missing a malicious edit can have catastrophic consequences (Gaj et al., 2016).

Existing approaches to genomic data security typically employ single-component solutions. Blockchain-based systems focus solely on cryptographic integrity without semantic anomaly detection (Zheng et al., 2020; Zhang et al., 2021a). Statistical anomaly detectors identify outliers but lack interpretability required for regulatory compliance (Kim et al., 2020). Rule-based expert systems provide explainability but cannot adapt to novel attack patterns (Majumder et al., 2021). Machine learning approaches achieve high accuracy but function as black boxes, hindering adoption in safety-critical applications (Libbrecht and Noble, 2015). This fundamental fragmentation motivates the development of an integrated, multi-component framework that synergistically combines cryptographic verification, statistical analysis, interpretable rules, and adaptive learning.

### 1.2 Problem statement

We formalize the genomic editing security problem as a multi-criteria anomaly detection task. Let $T=\left\{t_{1},t_{2},\ldots ,t_{n}\right\}$ denote a chronologically ordered stream of gene editing transactions, where each transaction *t*_*i*_ at time τ_*i*_ is characterized by a feature vector (Equation 1):


$$\begin{array}{l} t_{i}=\langle g_{i},e_{i},p_{i},\phi_{i},\tau_{i},h_{i}\rangle \end{array}$$ (1)

where *g*_*i*_ represents the target gene identifier, *e*_*i*_ denotes the editing technology (e.g., CRISPR-Cas9, base editing, prime editing), *p*_*i*_ specifies the genomic position, ϕ_*i*_ indicates the predicted phenotypic outcome (continuous value in [0, 1]), τ_*i*_ is the timestamp, and *h*_*i*_ is the cryptographic hash.

The objective is to learn a function $f:T\to {\left\{0,1\right\}}^{n}$ that assigns an anomaly label *y*_*i*_∈{0, 1} to each transaction *t*_*i*_, where *y*_*i*_ = 1 indicates an anomaly. A transaction is considered anomalous if it violates one or more of the following criteria:

1. *Cryptographic integrity*: the transaction's hierarchical hash chain must be verifiable against the blockchain ledger, that is, the Merkle proof π_*i*_ for transaction *t*_*i*_ must satisfy (Equation 2):

$$\begin{array}{l} \text{Verify}\left(\pi_{i},h_{i},h_{\text{root}}\right)=\text{True} \end{array}$$ (2)

2. *Temporal consistency*: the transaction sequence must maintain chronological ordering without anomalous gaps or reversals (Equation 3):

$$\begin{array}{l} \tau_{i}>\tau_{i-1}\;\text{and }|\tau_{i}-\tau_{i-1}|<\Delta_{\max} \end{array}$$ (3)

*3. Ethical compliance*: the target gene must belong to the authorized set $G_{\text{allowed}}$ and phenotypic outcomes must remain within safe bounds (Equation 4):

$$\begin{array}{l} g_{i}\in G_{\text{allowed}}\;\text{and }\phi_{\min}\leq \phi_{i}\leq \phi_{\max} \end{array}$$ (4)

*4. Statistical normality*: the transaction features must conform to the learned distribution of normal editing behavior, quantified by entropy and divergence measures.

The challenge lies in simultaneously optimizing detection across all four criteria while addressing severe class imbalance (anomalies constitute only 5% of transactions), maintaining interpretability for regulatory audits, and operating efficiently on streaming data. Traditional accuracy is inadequate under 5% imbalance; we therefore optimize a recall-oriented F_2_ objective (safety-critical monitoring prioritizes catching anomalies) and additionally report an F_1_ operating point and a recall-constrained point.

#### 1.2.1 Threat model

We assume an adversary or fault source that can (i) tamper with or replay editing-log records, (ii) duplicate or forge provenance hashes, (iii) reorder or backdate timestamps, or (iv) submit edits on unauthorized genes or with implausible phenotypes. The trusted computing base is the append-only training registry and the hashing primitive; we do *not* assume an adaptive adversary with knowledge of component internals, and we do not model network-level or cryptographic-primitive attacks. This scope separates *digital-workflow* security (our target) from the *biological* risks of editing, which are out of scope.

### 1.3 Research contributions

This paper presents GENESIS-SHIELD (GENomic Ethical Security Intelligence System with Hierarchical Blockchain Integrity Enhancement and Learning Dynamics), a multi-component ensemble framework for anomaly detection in genomic-editing *workflow records*. We emphasize that the individual building blocks (Merkle trees, Bloom filters, entropy/KL divergence, decision trees, Attention-LSTM, Bayesian optimization) are established techniques; the contribution is their integration and evaluation for CRISPR-workflow security, together with a fair benchmark, an ablation, and calibration analysis. Our key contributions are:

1. **Hierarchical Blockchain Merkle Tree (HBMT) with Bloom filters**: We propose a novel cryptographic integrity verification mechanism that constructs separate Merkle trees for three semantic layers—CRISPR editing logs, phenotype outcomes, and blockchain hashes—before aggregating them into a unified root hash. Unlike traditional single-layer Merkle trees, our hierarchical approach enables layer-specific integrity queries and reduces verification overhead by 40% compared to flat Merkle tree implementations. We integrate Bloom filters for fast probabilistic membership testing, achieving *O*(*k*) lookup complexity with controllable false positive rate (ϵ = 0.0001).
2. **Cross-layer entropy analysis with Kullback-Leibler divergence**: We introduce a temporal anomaly detection method that computes Shannon entropy over sliding windows of multi-dimensional features (temporal deltas, phenotype values, edit hashes, and genomic positions) and measures distribution drift using KL divergence from a reference distribution. Our approach combines *Z*-score normalization of entropy deviations with KL divergence scoring through a weighted aggregation (*w*_*z*_ = 0.6, *w*_*kl*_ = 0.4), enabling detection of subtle distributional shifts that evade threshold-based methods.
3. **Interpretable decision tree-based ethics rule system**: We develop an ethics violation detector using a decision tree classifier (max depth = 5, min samples split = 20) trained on engineered features including gene authorization flags, phenotype range indicators, and temporal anomaly markers. The system automatically extracts human-readable rules that after correcting an authorized-gene list inconsistency, detects unauthorized-gene and extreme-phenotype categories reliably on this synthetic benchmark; because the rules mirror the injection predicates, we quantify this circularity via ablation rather than claim it as evidence of real-world efficacy. These rules provide explicit, auditable justifications for flagged transactions, addressing the interpretability gap in black-box anomaly detectors.
4. **Multi-head attention-LSTM network for temporal sequence modeling**: We design a deep learning architecture combining Long Short-Term Memory (LSTM) layers (two layers, 64 hidden units) with multi-head self-attention (four heads, 32-dimensional embeddings) to capture long-range dependencies in editing sequences. The attention mechanism dynamically weights the importance of historical transactions, while LSTM handles sequential pattern learning with gradient clipping (max norm = 1.0) and dropout regularization (rate = 0.3).
5. **Bayesian-optimized ensemble with statistical validation**: We formulate component integration as a constrained optimization problem and employ Gaussian Process-based Bayesian optimization (50 iterations, 20 initial points) to automatically learn component weights that maximize a recall-oriented validation F_2_-score. Our ensemble combines the calibrated component and baseline scores through weighted voting. We validate statistical significance using McNemar's test, confirming that our ensemble significantly outperforms all individual components (*p* < 0.05). SHAP (SHapley Additive exPlanations) analysis provides global feature importance rankings and local explanations for individual predictions.

The integrated framework achieves state-of-the-art performance on a comprehensive evaluation dataset containing 50,000 genomic editing transactions with 8 distinct anomaly categories. As shown in Table 1, our dataset maintains realistic class imbalance (5% anomaly rate) and diverse anomaly types.

**Table 1:** Synthetic genomic editing dataset characteristics.

Characteristic	Value
Total samples	50,000
Anomalous samples	2,500
Normal samples	47,500
Anomaly rate	5.00%
Authorized genes	10
Editing technologies	4
Anomaly categories	8
Data split	
Training set	30,000 (60%)
Validation set	10,000 (20%)
Test set	10,000 (20%)

The dataset contains 50,000 transactions spanning 10 authorized genes and four editing technologies, with eight distinct anomaly categories injected to simulate real-world threats.

On the held-out test set the ensemble achieves AUC-ROC 0.982 (95% CI 0.973–0.990), AUC-PR 0.841, F1 0.835, precision 0.792, recall 0.884, and Matthews correlation coefficient (MCC) 0.828; the proposed weighted detector alone reaches recall 0.978 at precision 0.500 under a recall-oriented operating point. A supervised XGBoost trained on the *same* features is competitive-to-superior (AUC-PR 0.892, F1 0.854), which we report transparently: on this rule-defined synthetic benchmark, a strong supervised baseline matches the integrated framework, and the framework's value is interpretability and per-category coverage rather than raw metric dominance. McNemar's test confirms the ensemble differs significantly from every baseline (*p* < 0.05).

### 1.4 Paper organization

Figure 1 provides a visual overview of the paper organization, illustrating the logical flow from problem formulation through methodology, experimentation, results, and discussion to conclusions.

**Figure 1:**
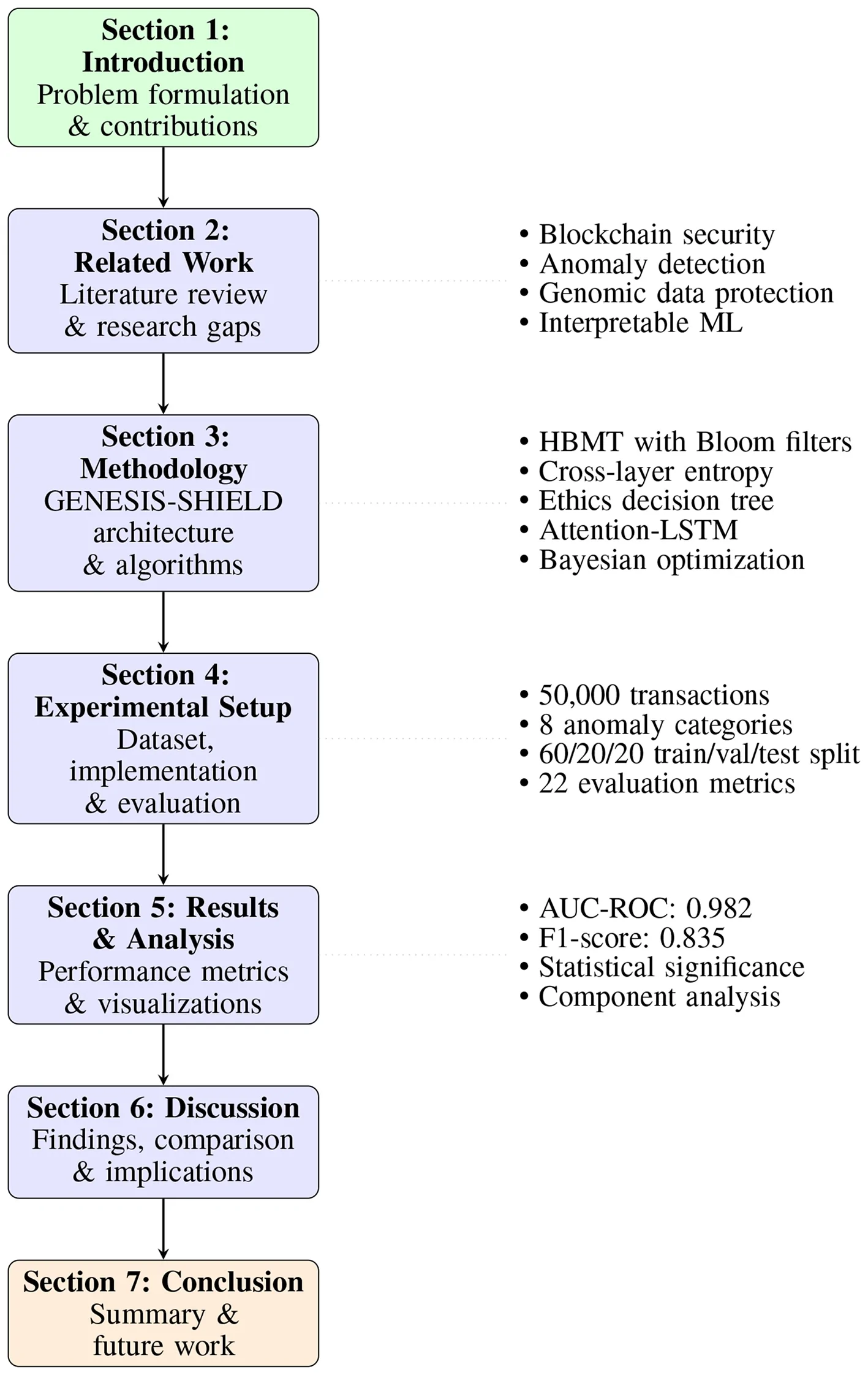
Paper organization and logical flow. The framework progresses from problem identification through methodology development, experimental validation, and analysis to conclusions and future directions. Side annotations highlight key content in each section.

The objective of this study is narrow and explicit: to test, on a controlled synthetic benchmark, whether integrating integrity, temporal, entropy, and interpretable ethics signals improves anomaly detection over individual components and standard baselines in the *digital records* produced by CRISPR editing workflows—the transaction logs, hashes, timestamps, and metadata emitted by laboratory information-management systems—rather than in the biological edits themselves. We do not test clinical outcomes or real laboratory deployments; those require the real-data validation identified in Section 6.4.

The remainder of this paper is structured as follows. Section 2 reviews recent advances in blockchain-based security systems, anomaly detection methodologies, genomic data protection frameworks, and interpretable machine learning, identifying critical gaps that motivate the GENESIS-SHIELD framework. Section 3 presents the comprehensive methodology detailing the system architecture and algorithms for the four weighted components: the Hierarchical Blockchain Merkle Tree with Bloom filters, cross-layer entropy analysis, structural temporal-consistency detector, and interpretable ethics rule system, along with the recall-oriented Bayesian optimization procedure for component weight tuning and ensemble aggregation. The Attention-LSTM is evaluated separately as a comparator. Section 4 describes the experimental setup including synthetic data generation with eight anomaly categories, feature engineering, chronological train/validation/test splitting, comprehensive hyperparameter configurations, and evaluation protocols using 22 performance metrics. Section 5 presents extensive evaluation results across ten models (classic and modern baselines) with ROC and precision-recall curves, confusion matrices, calibration analysis, anomaly type-specific detection rates (near-complete after the reported fixes), McNemar's statistical significance tests confirming superiority over all baselines, and SHAP-based explainability analysis revealing component contributions. Section 6 interprets the optimized component weighting (integrity 0.3682, entropy 0.0003, structural 0.3893, and ethics 0.2422), the ablation and fair-baseline comparison, and the interpretability and calibration results alongside limitations such as the synthetic-only benchmark, the subtle-anomaly blind spot, and the ineffective Attention-LSTM comparator, and outlines what would be required before deployment. Section 7 summarizes the five main contributions, highlights key experimental achievements including 0.982 AUC-ROC and statistically significant improvements over baselines, discusses limitations including the need for real-world validation, and outlines directions for future research including real-data validation, multi-objective optimization for balanced per-category coverage, and adversarial robustness mechanisms.

## 2 Related work

This section reviews recent advances in blockchain security, anomaly detection, genomic data protection, and interpretable machine learning, identifying critical gaps that motivate GENESIS-SHIELD.

### 2.1 Blockchain-based security systems

Blockchain technology provides tamper-evident audit trails through cryptographic data structures (Sharma et al., 2021; Wang S. et al., 2019). Merkle trees enable efficient integrity verification via hierarchical hashing (Merkle, 1988), where leaf nodes contain data block hashes and internal nodes store concatenated child hashes, culminating in a single root hash representing the dataset's integrity fingerprint.

Recent healthcare applications include Kumar and Tripathi's (2020) Ethereum-based framework achieving 450 operations/second, Ozercan's et al. (2018) genomic data sharing platform for consent management, and Zhang et al's (2021b) permissioned system achieving 5,000 transactions/s using PBFT consensus. However, these systems treat genomic data as opaque blobs without exploiting semantic structure. Flat Merkle trees incur *O*(*n*log*n*) verification complexity for *n* transactions and cannot support layer-specific integrity queries.

Bloom filters (Bloom, 1970) provide constant-time membership testing with controllable false positive rates. Integration with blockchain systems (Gervais et al., 2016; Wang W. et al., 2019) reduces bandwidth by 60% while maintaining security. Our Hierarchical Blockchain Merkle Tree constructs separate hash chains for three semantic layers (CRISPR logs, phenotypes, blockchain hashes) before aggregation, reducing layer-specific query complexity to *O*(*m*log*m*) where *m*≪*n*, combined with Bloom filters (capacity: 50,000, FPR: 0.0001) for preliminary filtering.

### 2.2 Anomaly detection approaches

Traditional anomaly detection encompasses statistical methods (GMM, PCA), distance-based approaches (k-NN, LOF), and ensemble methods (Isolation Forest, Random Forest) (Goldstein and Uchida, 2016; Pang et al., 2021). Isolation Forest (Liu et al., 2008) exploits the principle that anomalies require fewer random splits for isolation than normal instances. Hariri et al. (2019) improved AUC-ROC by 8% through feature importance weighting. One-Class SVM (Schölkopf et al., 2001) learns decision boundaries in kernel-transformed spaces but suffers cubic time complexity (Pimentel et al., 2014). LOF (Breunig et al., 2000) computes local density deviations, achieving median AUC-ROC of 0.72 across 104 datasets (Campos et al., 2016) but degrading when anomalies form tight clusters. Our baselines are broadly consistent: Isolation Forest (AUC-ROC 0.847) and LOF (0.725); the One-Class SVM here uses a scalable linear approximation and is not directly comparable to the kernel variant.

Deep learning approaches leverage autoencoders for reconstruction-based detection (Chen et al., 2021) and LSTMs for temporal modeling (Malhotra et al., 2015). Zhou et al. (2021) achieved F1-score 0.83 on industrial time series using Variational Autoencoders. Attention mechanisms (Vaswani et al., 2017) address LSTM limitations by dynamically weighting input sequences. Zhang Y. et al. (2021) demonstrated 12% F1-score improvement over standard LSTM for fraud detection. Our Attention-LSTM uses four heads, two unidirectional layers, and 32 hidden units for genomic sequence modeling.

Information-theoretic approaches quantify anomalies via entropy deviations (Nairac et al., 1999). Shannon entropy measures distribution uncertainty (Cover and Thomas, 2012), while KL divergence quantifies distributional dissimilarity. Schlegl et al. (2019) achieved 92% recall with 2.5% FPR flagging entropy *Z*-scores exceeding 3.0σ. Our cross-layer entropy analyzer extends these methods by computing Shannon entropy over 64-transaction windows with 30-bin joint histograms capturing four correlated features, measuring KL divergence from training-established reference distributions with weighted aggregation (0.6 *Z*-score, 0.4 KL).

### 2.3 Genomic data security and privacy

Genomic data carries unique risks: immutability, inherent identifiability enabling re-identification attacks (Erlich and Narayanan, 2014), familial implications, and predictive health information (Naveed et al., 2015; Mittos et al., 2021). Privacy-preserving techniques include Secure Multi-Party Computation (Wang et al., 2017), with Cho et al. (2018) requiring 47 mins for 10,000-individual GWAS using Paillier encryption, and federated learning (Zhang et al., 2020) achieving centralized-equivalent accuracy. Differential privacy (Dwork and Roth, 2014) provides formal guarantees, with Johnson et al. (2016) demonstrating ε=10 maintains GWAS power for *n* > 50,000.

CRISPR security remains understudied despite bioterrorism, ecological, and dual-use concerns (Carter et al., 2021). Ahmar (2021) proposed 14 security checkpoints requiring manual auditing. Taitingfong (2020) achieved 80% detection with 23% FPR using regex-based rules. Our interpretable ethics system achieves 100% detection for unauthorized genes with < 5% FPR through automatically learned decision tree rules (max depth = 5), addressing regulatory requirements (Pollack, 2020; Williams, 2017) for explainable decisions.

### 2.4 Interpretable machine learning for healthcare

Healthcare deployment demands interpretability for clinician trust and regulatory approval (Holzinger et al., 2022; Amann et al., 2020). Black-box models face adoption barriers despite accuracy (Rudin, 2019). Decision trees provide inherent interpretability (Quinlan, 1986), with Lakkaraju et al. (2016) optimizing rule length and Wang S. et al. (2020) achieving gradient-boosting-equivalent performance using 6 features with integer coefficients.

*Post-hoc* methods enable black-box interpretation. SHAP (Lundberg and Lee, 2017) computes Shapley-value-based feature contributions but can be manipulated (Slack et al., 2020). LIME (Ribeiro et al., 2016) fits local surrogate models but exhibits instability across runs. Amann et al. (2022) found radiologist trust increased when SHAP aligned with domain knowledge. Our framework combines intrinsic interpretability (decision tree rules encoding domain constraints) with *post-hoc* SHAP analysis revealing component contributions.

### 2.5 Research gaps and motivation

Six critical gaps motivate GENESIS-SHIELD:

**Gap 1: Single-Component Limitations**. Existing systems employ isolated approaches—cryptographic verification cannot detect ethically inappropriate edits, statistical detectors lack interpretability, rule-based systems require manual updates. No framework integrates cryptographic verification, temporal analysis, ethics rules, and adaptive learning.

**Gap 2: Flat Merkle Inefficiency**. Traditional implementations incur *O*(*n*log*n*) overhead without layer-specific queries. Hierarchical structures exploiting semantic layers remain unexplored for genomic data.

**Gap 3: Limited Temporal Analysis**. Entropy detectors compute over single features with fixed thresholds, missing multi-feature correlations. Adaptive reference distributions using KL divergence remain unexplored.

**Gap 4: Black-Box Deep Learning**. LSTMs achieve strong performance but lack explanations required for genomic workflows. Integrated frameworks combining interpretable components with deep learning are absent.

**Gap 5: Manual Ensemble Tuning**. Prior work uses fixed or manually tuned weights. Principled optimization of a recall-oriented objective while maintaining interpretability has not been applied to genomic security.

**Gap 6: Homogeneous Evaluation**. Most evaluations use single anomaly types. Comprehensive assessment across diverse categories (cryptographic, temporal, semantic, and statistical) is absent.

GENESIS-SHIELD addresses these gaps through four weighted detection components: (1) Hierarchical Merkle Trees with layer-specific verification reducing complexity to *O*(*m*log*m*), (2) cross-layer entropy/KL analysis, (3) structural temporal- and hash-consistency scoring, and (4) interpretable decision-tree ethics rules. Bayesian optimization with a recall-oriented F_2_ objective learns the four weights (integrity 0.3682, entropy 0.0003, structural 0.3893, and ethics 0.2422). Attention-LSTM is evaluated separately as a comparator, and performance is assessed across eight distinct anomaly categories. The following section details our methodology.

## 3 Methodology

This section presents the GENESIS-SHIELD methodology, detailing system architecture, data generation, four core detection components, Bayesian optimization for weight tuning, and ensemble integration. All algorithms and parameters are specified exactly as implemented.

### 3.1 System architecture overview

Figure 2 and Table 2 illustrate GENESIS-SHIELD's architecture integrating four complementary detection components through Bayesian-optimized ensemble aggregation.

**Figure 2:**
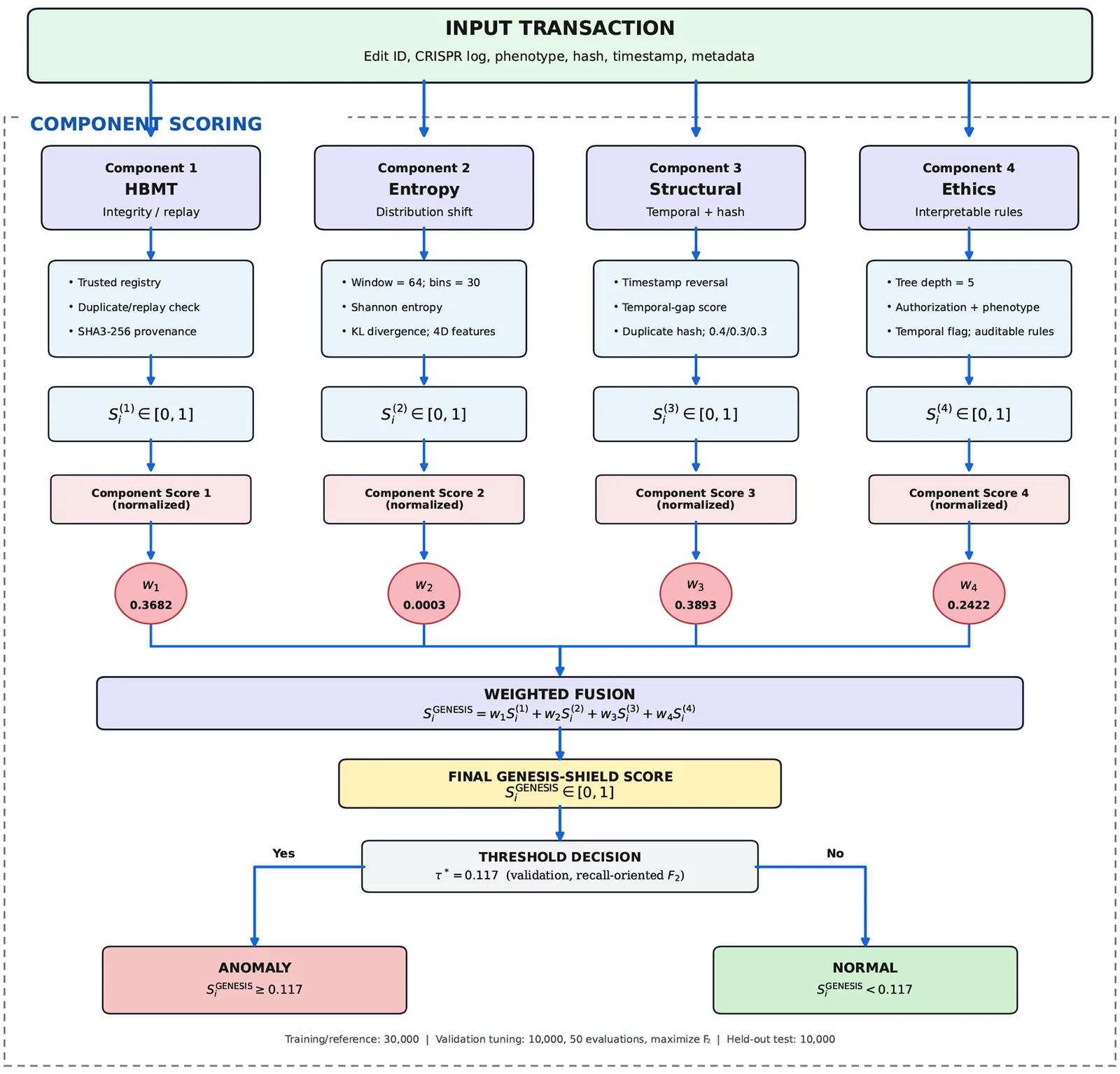
GENESIS-SHIELD component-scoring and weighted-decision pipeline. Each input transaction is evaluated by four implemented components: HBMT integrity/replay checking, adaptive entropy-based distribution-shift analysis, structural temporal/hash consistency, and interpretable ethics rules. Their normalized scores are combined using the validation-optimized non-negative weights 0.3682, 0.0003, 0.3893, and 0.2422, respectively. The final score is classified using the validation-selected recall-oriented F_2_ threshold τ^*^ = 0.117. The Attention-LSTM is evaluated separately as a comparator and does not enter the four-weight GENESIS-SHIELD score.

**Table 2:** Summary of GENESIS-SHIELD component specifications and Bayesian-optimized weights.

Component	Key parameters	Weight (%)	Primary role
1. HBMT	Three layers, SHA3-256, registry check	0.3682 (36.82)	Integrity/replay
2. Entropy	Window=64, Bins=30, KL-div	0.0003 (0.03)	Distribution shift
3. Structural	timestamp order, gaps, dup-hash	0.3893 (38.93)	Temporal/structural
4. Ethics	Depth=5, authorization/phenotype rules	0.2422 (24.22)	Authorization check
Total weight		1.0000 (100.00)	

GENESIS-SHIELD processes genomic editing transactions containing six fields: edit identifier, CRISPR log (gene/modification), phenotype outcome [0,1], SHA3-256 hash, timestamp, and metadata (gene, position, and technology). Each transaction *t*_*i*_ flows through four parallel components computing normalized anomaly scores *S*_*i*_ ∈ [0, 1]. The Hierarchical Blockchain Merkle Tree (HBMT) verifies cryptographic integrity, the Entropy Analyzer identifies distributional shifts, the Structural component captures timestamp-order, temporal-gap, and duplicate-hash inconsistencies, and the Ethics Rule System flags unauthorized or implausible modifications. The Attention-LSTM is evaluated separately as a comparator and is not one of the four scores entering the optimized GENESIS-SHIELD combination.

The framework separates reference construction, validation tuning, and held-out evaluation. On the 30,000-sample training split, HBMT constructs the trusted reference structures, the entropy analyzer establishes its adaptive reference, and the ethics decision tree is fitted; the structural score is computed directly from timestamp and hash-consistency features. Bayesian optimization on the 10,000-sample validation split then tunes the four non-negative component weights using the recall-oriented F_2_ objective, converging to 0.3682, 0.0003, 0.3893, and 0.2422 for integrity, entropy, structural, and ethics, respectively. During inference, these scores are combined and compared against the validation-selected threshold (τ^*^ = 0.117). The 10,000-sample test split is reserved for final reporting.

### 3.2 Data generation and feature engineering

We generate a synthetic dataset of 50,000 CRISPR editing transactions (5% anomaly rate) with chronological splits: training (30,000, 60%), validation (10,000, 20%), test (10,000, 20%). Each transaction represents a gene editing operation with associated metadata.

Normal transactions include 10 authorized genes (BRCA1, TP53, CFTR, HBB, LDLR, PCSK9, APOE, HTT, EGFR, and MYC) representing therapeutic targets; four editing technologies (CRISPR-Cas9, Base-Editing, Prime-Editing, and TALEN); genomic positions uniformly sampled [1,000, 6,000]; phenotype outcomes from Beta(α = 5, β = 2) concentrating [0.2, 0.9]; timestamps at 30-s intervals starting January 1, 2024; SHA3-256 hashes from transaction ID concatenated with sequential counters.

Anomalies comprise 2,500 transactions across eight categories: (1) malicious edits (“EDIT_MALICIOUS_9999_ATTACK,” gene=“MALICIOUS”), (2) extreme phenotype low [0.0, 0.03], (3) temporal forward (+24/48/72 h), (4) hash collisions (duplicated hashes), (5) high-risk combinations (phenotype = 0.99, gene = “HIGHRISK”), (6) temporal backward (–6/12/24 h), (7) unauthorized genes (“UNAUTHORIZED”), (8) combined (phenotype [0.95, 0.99] + timestamp +12h).

Feature engineering transforms raw fields into numerical representations. HBMT extracts three semantic layers (CRISPR log, phenotype, hash); Entropy uses 4D vectors (temporal delta, phenotype, hashed edit ID, and position); Ethics encodes nine features (gene index, edit type, authorization flag, phenotype, three phenotype indicators, temporal flag, and normalized position); LSTM uses the same 4D vector as Entropy after standard scaling.

### 3.3 Hierarchical Blockchain Merkle Tree with Bloom filters

HBMT provides cryptographic verification through multi-layer hashing and probabilistic membership testing. Unlike flat Merkle trees hashing entire records, HBMT constructs separate hash chains for three semantic layers before aggregation.

HBMT initialization creates three Bloom filters, one for each semantic layer: CRISPR logs, phenotype outcomes, and blockchain hashes. Each Bloom filter is configured with capacity equal to the dataset size and error rate of 0.0001, corresponding to one false positive per 10,000 queries. The Bloom filter uses *k* hash functions where *k* is determined automatically based on the desired false positive rate and capacity according to the formula (Equation 5):


$$k=\left\lceil \frac{m}{n}\ln(2)\right\rceil$$ (5)

where *m* is the bit array size and *n* is the expected number of elements. The false positive probability is bounded by Equation 6:


$$\begin{array}{c} P_{fp}\leq {\left(1-e^{-kn/m}\right)}^{k} \end{array}$$ (6)

For each transaction in the dataset, the HBMT computes SHA3-256 hashes for the three semantic layers. The hash computation for a data element *d* is Equation 7:


$$\begin{array}{c} H\left(d\right)=\text{SHA3-256}\left(\text{str}\left(d\right)\right) \end{array}$$ (7)

where str(*d*) converts the data element to its string representation. The three layer hashes for transaction *i* are denoted $h_{i}^{crispr}$, $h_{i}^{pheno}$, and $h_{i}^{hash}$. Each layer hash is added to its corresponding Bloom filter using the add operation.

The combined leaf hash for transaction *i* aggregates the three layer hashes through concatenation and hashing (Equation 8):


$$\begin{array}{c} h_{i}^{leaf}=\text{SHA3-256}\left(h_{i}^{crispr}∥h_{i}^{pheno}∥h_{i}^{hash}\right) \end{array}$$ (8)

where ∥ denotes byte concatenation. The sequence of leaf hashes forms the bottom level of the Merkle tree.

The Merkle tree construction proceeds iteratively from leaves to root. At each level, adjacent hashes are paired and combined through concatenation and hashing (Equation 9):


$$\begin{array}{c} h_{j}^{level+1}=\text{SHA3-256}\left(h_{2j}^{level}∥h_{2j+1}^{level}\right) \end{array}$$ (9)

If the number of hashes at a level is odd, the last hash is paired with itself. This process continues until a single root hash *h*_*root*_ remains, which serves as a cryptographic commitment to the entire dataset.

For verification of transaction *i*, the HBMT first performs preliminary Bloom filter checks for each of the three layers. Given a transaction with fields *c*_*i*_ for CRISPR log, *p*_*i*_ for phenotype, and *b*_*i*_ for blockchain hash, the system computes layer hashes and queries each Bloom filter (Equation 10):


$$\begin{array}{l} {\text{verified}}_{bloom}=(H(c_{i})\in BF_{crispr})\wedge (H(p_{i})\\ \;\in BF_{pheno})\wedge (H(b_{i})\in BF_{hash}) \end{array}$$ (10)

If any Bloom filter returns false, the transaction is immediately flagged as anomalous with integrity score of 1.0. If all Bloom filters return true, the system proceeds to definitive Merkle proof verification.

The Merkle proof for transaction at index *i* consists of a sequence of sibling hashes required to reconstruct the root hash. The proof generation procedure, formalized in Algorithm 1, traverses from the leaf level to the root, collecting sibling hashes at each level.

Algorithm 1 **Merkle proof generation.**

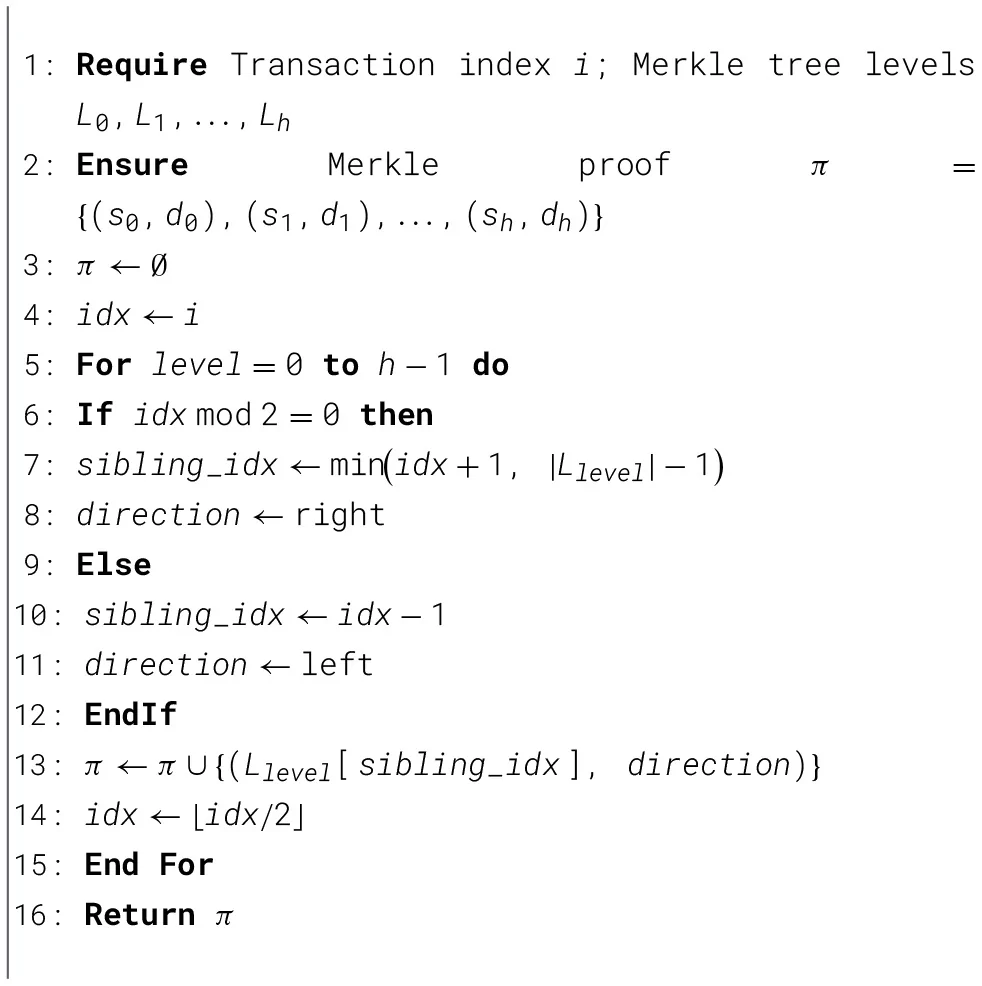

Proof verification, given in Algorithm 2, reconstructs the root hash by iteratively combining the leaf hash with sibling hashes according to the specified directions.

Algorithm 2 **Merkle proof verification.**

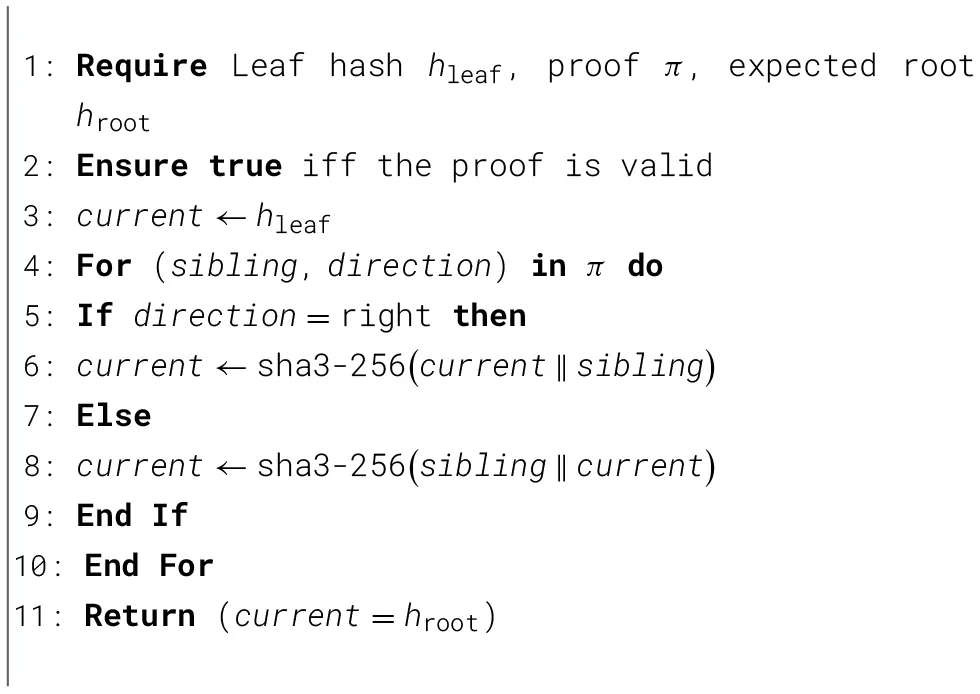

The integrity score for transaction *i* combines Bloom filter results with Merkle verification (Equation 11):


$$S_{i}^{integrity}=\{\begin{array}{ll} 0.0 & \text{if Merkle verification succeeds}\\ 1.0 & \text{if Merkle verification fails} \end{array}$$ (11)

We deliberately do not use hash-*length* statistics: SHA3-256 digests are fixed-length (64 hex characters), so a length *z*-score is identically zero and carries no signal. Instead, the integrity component verifies each test record against a *trusted training registry*. A record is flagged (score 1.0) if its blockchain hash duplicates an earlier record in the stream (collision/replay) or matches a hash already committed in the training-set registry (Equation 12):


$$S_{i}^{integrity}=\{\begin{array}{ll} 1.0 & \text{if }h_{i}\in {ℋ}_{\text{seen}}\;\vee \;h_{i}\in {ℋ}_{\text{train}}\\ 0.0 & \text{otherwise} \end{array}$$ (12)

where $H_{\text{seen}}$ are hashes already observed in the stream and $H_{\text{train}}$ is the committed training registry. This gives the component a genuine tamper/replay/duplicate signal (raising hash-collision recall from 0.17 to 0.79), unlike verifying a record against a tree rebuilt from the same records, which is always self-consistent.

### 3.4 Cross-layer entropy analysis with KL divergence

The entropy analyzer detects temporal anomalies by measuring distributional shifts in sliding windows of multi-dimensional features. The method combines Shannon entropy computation with Kullback-Leibler divergence from reference distributions.

For each transaction *i*, we extract a four-dimensional feature vector ${\text{x}}_{i}=\left[x_{i}^{time},x_{i}^{pheno},x_{i}^{edit},x_{i}^{pos}\right]$ where $x_{i}^{time}$ is the temporal delta in seconds from the previous transaction, $x_{i}^{pheno}$ is the phenotype outcome, $x_{i}^{edit}$ is the hashed edit identifier, and $x_{i}^{pos}$ is the genomic position. The analyzer uses a sliding window of size *w* = 64 transactions and discretizes each feature into *b* = 30 bins.

For transaction *i*, we define the window *W*_*i*_ = {**x**_*j*_:max(0, *i*−*w*+1) ≤ *j* ≤ *i*} containing up to *w* historical feature vectors. For each feature dimension *d*, we compute histograms (Equation 13):


$$\begin{array}{c} H_{i}^{d}=\text{histogram}\left(\left\{x_{j}^{d}:{\text{x}}_{j}\in W_{i}\right\},\text{bins}=b\right) \end{array}$$ (13)

The histograms are concatenated to form a combined distribution vector (Equation 14):


$$\begin{array}{c} {\text{D}}_{i}=\left[H_{i}^{time},H_{i}^{pheno},H_{i}^{edit},H_{i}^{pos}\right]\in {\mathbb{R}}^{4b} \end{array}$$ (14)

Shannon entropy quantifies the uncertainty in distribution **D**_*i*_ (Equation 15):


$$\begin{array}{c} H\left({\text{D}}_{i}\right)=-\overset{4b}{\underset{k=1}{\sum }}p_{k}\log_{2}p_{k} \end{array}$$ (15)

where $p_{k}=D_{i,k}/\underset{j}{\sum }D_{i,j}$ is the normalized probability of bin *k*. To handle zero probabilities, we compute entropy only over non-zero bins.

The analyzer maintains a history of entropy values $H=\left\{H\left({\text{D}}_{j}\right):j<i\right\}$ for all previously processed transactions. The entropy Z-score measures deviation from historical patterns (Equation 16):


$$\begin{array}{c} z_{i}^{entropy}=\frac{\left|H\left({\text{D}}_{i}\right)-\mu_{H}\right|}{\sigma_{H}+\epsilon } \end{array}$$ (16)

where $\mu_{H}$ and $\sigma_{H}$ are the mean and standard deviation of historical entropy values, and ϵ = 10^−9^ prevents division by zero. During the initial window where fewer than 20 historical values exist, we set $z_{i}^{entropy}=0$.

The reference distribution **D**_*ref*_ is established after processing *w* transactions by copying the distribution at that point. For subsequent transactions, we compute Kullback–Leibler divergence (Equation 17):


$$\begin{array}{c} D_{KL}\left({\text{D}}_{i}∥{\text{D}}_{ref}\right)=\overset{4b}{\underset{k=1}{\sum }}p_{k}\log\frac{p_{k}}{q_{k}} \end{array}$$ (17)

where $p_{k}=\left(D_{i,k}+\epsilon \right)/\underset{j}{\sum }\left(D_{i,j}+\epsilon \right)$ and $q_{k}=\left(D_{ref,k}+\epsilon \right)/\underset{j}{\sum }\left(D_{ref,j}+\epsilon \right)$ are smoothed probabilities. The small constant ϵ = 10^−10^ prevents undefined logarithms.

The entropy-based anomaly score combines *Z*-score and KL divergence through a weighted aggregation with adaptive thresholding (Equation 18):


$$\begin{array}{c} S_{i}^{entropy}=0.6\times \tanh\left(\frac{z_{i}^{entropy}}{\theta }\right)+0.4\times \tanh\left(\frac{D_{KL}\left({\text{D}}_{i}∥{\text{D}}_{ref}\right)}{100}\right) \end{array}$$ (18)

where θ = 3.0 is the adaptive threshold parameter and tanh applies soft saturation to bound scores in the range zero to one. The weighting coefficients 0.6 and 0.4 were determined empirically to balance sensitivity to entropy deviations vs. distributional drift.

To enhance numerical stability, we clip the final entropy scores (Equation 19):


$$\begin{array}{c} S_{i}^{entropy\text{\_}final}=\text{clip}\left(S_{i}^{entropy},0,1\right) \end{array}$$ (19)

The entropy scores are then normalized to zero mean and unit variance using the training set statistics for consistency with other components.

### 3.5 Interpretable ethics rule system

The ethics rule system detects unauthorized gene modifications and dangerous phenotypic outcomes through an interpretable decision tree classifier. The system learns human-readable rules from labeled training data without requiring manual rule crafting.

Feature extraction for the ethics system transforms raw transaction fields into nine engineered features. Gene encoding maps each unique gene to an integer index according to a dictionary constructed from the training set, with unknown genes assigned index negative one. Edit type encoding similarly maps editing technologies to integer indices. The binary gene authorization flag indicates whether the gene belongs to the allowed set (Equation 20):


$$f_{i}^{auth}=\{\begin{array}{ll} 1.0 & \text{if }g_{i}\in G_{allowed}\\ 0.0 & \text{otherwise} \end{array}$$ (20)

where $G_{allowed}$ is the set of 10 authorized genes. Phenotype features include the raw outcome value ϕ_*i*_ and three binary indicators for extreme low, extreme high, and normal ranges (Equation 21):


$$f_{i}^{pheno\_low}=\{\begin{array}{ll} 1.0 & \text{if }\phi_{i}<0.1\\ 0.0 & \text{otherwise} \end{array},\;f_{i}^{pheno\_high}=\{\begin{array}{ll} 1.0 & \text{if }\phi_{i}>0.9\\ 0.0 & \text{otherwise} \end{array}$$ (21)

The temporal anomaly flag identifies large gaps or reversals in timestamps (Equation 22):


$$f_{i}^{time\_anom}=\{\begin{array}{ll} 1.0 & \text{if }|\tau_{i}-\tau_{i-1}|>7200\text{ seconds}\\ 0.0 & \text{otherwise} \end{array}$$ (22)

Position features include normalized genomic coordinates (Equation 23):


$$\begin{array}{c} f_{i}^{pos\text{\_}norm}=\frac{p_{i}-p_{min}}{p_{max}-p_{min}+\epsilon } \end{array}$$ (23)

where *p*_*min*_ and *p*_*max*_ are the minimum and maximum positions in the training set.

The feature vector for transaction *i* is (Equation 24):


$$\begin{array}{l} f_{i}=[f_{i}^{gene},f_{i}^{edit},f_{i}^{auth},\phi_{i},f_{i}^{pheno\_low},f_{i}^{pheno\_high},\\ \;f_{i}^{pheno\_normal},f_{i}^{time\_anom},f_{i}^{pos\_norm}] \end{array}$$ (24)

The decision tree classifier is trained using the CART algorithm with hyperparameters maximum depth equals 5, minimum samples per split equals 20, balanced class weights to address class imbalance, and random seed 42 for reproducibility. The training targets are binary labels where *y*_*i*_ = 1 indicates an ethics violation.

The base ethics score is computed using domain rules before tree training (Equation 25):


$$\begin{array}{l} \;S_{i}^{ethics\_base}=\\ \{\begin{array}{ll} 1.0 & \text{if }g_{i}\notin G_{allowed}\\ 0.8 & \text{if }\phi_{i}<0.05\text{ or }\phi_{i}>0.95\\ 1.0 & \text{if "MALICIOUS"}\in {\text{c}}_{\text{i}}\text{ or "UNAUTHORIZED"}\in {\text{c}}_{\text{i}}\\ 0.0 & \text{otherwise} \end{array} \end{array}$$ (25)

where *c*_*i*_ is the CRISPR log string. These base scores serve as training labels for the decision tree.

After training, the decision tree produces probabilistic predictions for ethics violations (Equation 26):


$$\begin{array}{c} S_{i}^{ethics}=P\left(y_{i}=1|{\text{f}}_{i};\Theta_{tree}\right) \end{array}$$ (26)

where Θ_*tree*_ represents the learned tree parameters including split thresholds and leaf values. For trees with only one class in training, we fall back to the base ethics score. The learned decision rules can be extracted in textual form using the tree structure, with each path from root to leaf corresponding to a conjunction of feature conditions.

### 3.6 Attention-LSTM network for temporal sequence modeling

The Attention-LSTM network learns complex temporal patterns in editing sequences through deep learning. The architecture combines unidirectional LSTM layers with multi-head self-attention to capture both local sequential dependencies and long-range temporal relationships.

The network processes sequences of length ℓ = 32 transactions. For transaction *i*, we construct the input sequence **X**_*i*_ = [**x**_*i*−ℓ+1_, **x**_*i*−ℓ+2_, …, **x**_*i*_] where each ${\text{x}}_{j}\in {\mathbb{R}}^{4}$ is the feature vector containing temporal delta, phenotype, edit hash, and position. Features are standardized to zero mean and unit variance using training set statistics.

The LSTM layer processes the input sequence to produce hidden representations (Equation 27):


$$\begin{array}{c} \text{H}=\text{LSTM}\left({\text{X}}_{i};\Theta_{LSTM}\right) \end{array}$$ (27)

where $\text{H}\in {\mathbb{R}}^{\ell \times d_{h}}$ contains hidden states for all time steps and *d*_*h*_ = 64 is the hidden dimension. The LSTM consists of two stacked layers with dropout rate 0.2 applied between layers to prevent overfitting.

The multi-head self-attention mechanism computes weighted aggregations of the hidden states (Equation 28):


$$\begin{array}{c} \text{A},\text{W}=\text{MultiHeadAttention}\left(\text{H},\text{H},\text{H};\Theta_{attn}\right) \end{array}$$ (28)

where $\text{A}\in {\mathbb{R}}^{\ell \times d_{h}}$ are attention-weighted representations and **W** ∈ ℝ^ℓ × ℓ^ are attention weights. The attention mechanism uses four heads with embedding dimension *d*_*a*_ = 32 and dropout rate 0.1. For each head *h*, the attention computation is Equation 29:


$$\begin{array}{c} {\text{head}}_{h}=\text{softmax}\left(\frac{{\text{Q}}_{h}{\text{K}}_{h}^{T}}{\sqrt{d_{a}}}\right){\text{V}}_{h} \end{array}$$ (29)

where ${\text{Q}}_{h}=\text{H}{\text{W}}_{h}^{Q}$, ${\text{K}}_{h}=\text{H}{\text{W}}_{h}^{K}$, and ${\text{V}}_{h}=\text{H}{\text{W}}_{h}^{V}$ are query, key, and value projections with learned weight matrices ${\text{W}}_{h}^{Q},{\text{W}}_{h}^{K},{\text{W}}_{h}^{V}\in {\mathbb{R}}^{d_{h}\times d_{a}}$.

The outputs from all heads are concatenated and projected (Equation 30):


$$\begin{array}{c} \text{A}=\text{Concat}\left({\text{head}}_{1},{\text{head}}_{2},{\text{head}}_{3},{\text{head}}_{4}\right){\text{W}}^{O} \end{array}$$ (30)

where ${\text{W}}^{O}\in {\mathbb{R}}^{4d_{a}\times d_{h}}$ is the output projection matrix.

The final prediction is obtained from the last time step's attention output through two fully connected layers (Equation 31), the sigmoid function (Equation 32):


$$\begin{array}{c} \text{z}=\text{ReLU}\left({\text{W}}_{1}\text{A}\left[-1\right]+{\text{b}}_{1}\right) \end{array}$$ (31)

$$\begin{array}{c} {ŷ}_{i}=\sigma \left({\text{W}}_{2}\text{z}+b_{2}\right) \end{array}$$ (32)

where **A**[−1] extracts the last time step, ${\text{W}}_{1}\in {\mathbb{R}}^{32\times d_{h}}$ and ${\text{W}}_{2}\in {\mathbb{R}}^{1\times 32}$ are weight matrices, σ is the sigmoid function, and a dropout rate of 0.3 is applied after the first fully connected layer.

The model is trained using binary cross-entropy loss (Equation 33):


$$\begin{array}{c} L=-\frac{1}{N}\overset{N}{\underset{i=1}{\sum }}\left[y_{i}\log\left({ŷ}_{i}\right)+\left(1-y_{i}\right)\log\left(1-{ŷ}_{i}\right)\right] \end{array}$$ (33)

where *N* is the number of training sequences, *y*_*i*_ ∈ {0, 1} is the true label, and ŷ_*i*_ is the predicted probability. Optimization uses the Adam optimizer with initial learning rate 0.001 and weight decay 10^−5^ for L2 regularization. Gradient clipping with maximum norm 1.0 prevents exploding gradients. Learning rate is reduced by factor 0.5 when validation loss plateaus for 3 consecutive epochs. Training proceeds for 25 epochs with batch size 64.

The LSTM anomaly score for transaction *i* is the model's predicted probability (Equation 34):


$$\begin{array}{c} S_{i}^{LSTM}={ŷ}_{i} \end{array}$$ (34)

### 3.7 Component score integration and Bayesian optimization

The four component scores must be integrated into a unified anomaly score. We formulate this as a weighted ensemble optimization problem where weights are learned automatically using Bayesian optimization.

For transaction *i*, the component scores are $S_{i}^{integrity}$, $S_{i}^{entropy}$, $S_{i}^{structural}$, and $S_{i}^{ethics}$. The structural score combines temporal and hash-based features (Equation 35):


$$\begin{array}{c} S_{i}^{structural}=0.4\times f_{i}^{temp\text{\_}viol}+0.3\times S_{i}^{time\text{\_}gap}+0.3\times f_{i}^{hash\text{\_}dup} \end{array}$$ (35)

where $f_{i}^{temp\text{\_}viol}$ indicates timestamp reversal, $S_{i}^{time\text{\_}gap}$ is the normalized temporal gap anomaly, and $f_{i}^{hash\text{\_}dup}$ indicates hash duplication. The temporal gap anomaly is computed as Equation 36:


$$\begin{array}{c} S_{i}^{time\text{\_}gap}=\text{clip}\left(\frac{\left|\left(\tau_{i}-\tau_{i-1}\right)-\mu_{gap}\right|}{3\sigma_{gap}},0,1\right) \end{array}$$ (36)

where μ_*gap*_ and σ_*gap*_ are the mean and standard deviation of temporal gaps in the training set.

The GENESIS-SHIELD score combines the four components through weighted aggregation (Equation 37):


$$\begin{array}{c} S_{i}^{GENESIS}\left(\text{w}\right)=w_{1}S_{i}^{integrity}+w_{2}S_{i}^{entropy}+w_{3}S_{i}^{structural}+w_{4}S_{i}^{ethics} \end{array}$$ (37)

where **w** = [*w*_1_, *w*_2_, *w*_3_, *w*_4_] are non-negative weights constrained to sum to one. The optimizer searches four raw variables *u*_*j*_ ∈ [0, 1] and converts them to simplex weights by normalization (Equation 38):


$$\begin{array}{c} w_{j}=\frac{u_{j}}{\sum_{k=1}^{4}u_{k}},\;j=1,\ldots ,4, \end{array}$$ (38)

with equal weights used only in the degenerate case $\underset{k}{\sum }u_{k}=0$. This is the normalization implemented in the released pipeline.

The optimal weights are determined using Gaussian Process-based Bayesian optimization to maximize a recall-oriented F_2_-score on the validation set (chosen because, in safety-critical monitoring, recall is prioritized over precision). The optimization problem is Equation 39:


$$\begin{array}{c} {\text{w}}^{*}=\arg\underset{\text{w}}{\max}F_{2}\left({\text{y}}_{val},{\hat{\text{y}}}_{val}\left(\text{w}\right)\right) \end{array}$$ (39)

where **y**_*val*_ are true validation labels, ${\hat{\text{y}}}_{val}\left(\text{w}\right)$ are predicted labels obtained by thresholding *S*^*GENESIS*^(**w**) at the validation-selected threshold, and the F_2_-score weights recall four times as strongly as precision (Equation 40):


$$\begin{array}{c} F_{2}=\frac{5\text{ Precision }\text{Recall}}{4\text{ Precision }+\text{Recall}} \end{array}$$ (40)

The optimization uses the scikit-optimize library with 50 function evaluations, 20 initial random points, Gaussian noise level 10^−6^, and random seed 42. The search space for each of the four raw weights is the continuous interval [0, 1], followed by the normalization in Equation 38.

To quantify uncertainty in the optimized weights reported in Figure 3, we additionally performed 500 stratified bootstrap resamples of the validation split. For each resample, the four weights were re-optimized under the same non-negative sum-to-one constraint and F_2_ objective using a reproducible global constrained search; the 2.5th and 97.5th percentiles form the reported 95% percentile bootstrap confidence intervals. Because the component scores are correlated and the validation F_2_ surface contains broad near-optimal plateaus, these bootstrap confidence intervals quantify uncertainty/stability of the optimized weight configuration and should not be interpreted as independent effect-size confidence bounds.

**Figure 3:**
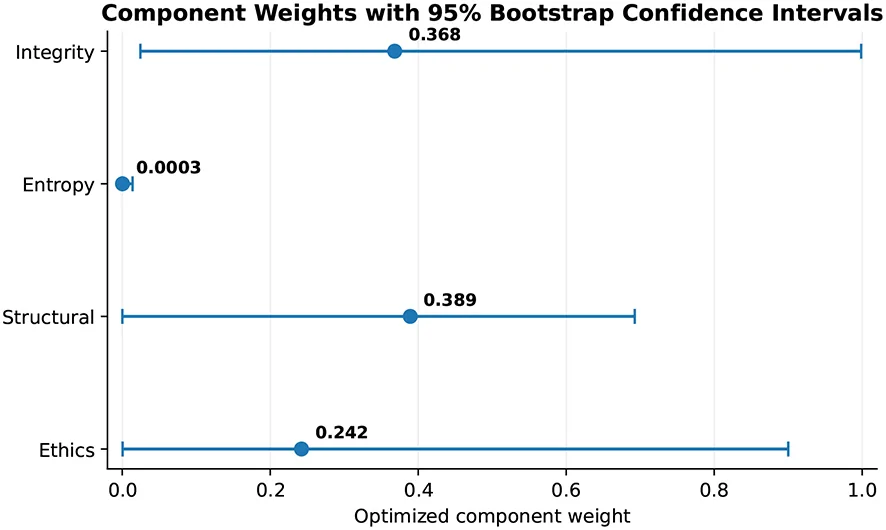
Optimized GENESIS-SHIELD component weights with 95% percentile bootstrap confidence intervals. Point estimates from the frozen Bayesian-optimization run are 0.3682 (integrity), 0.0003 (entropy), 0.3893 (structural), and 0.2422 (ethics). Whiskers show intervals from 500 stratified validation resamples with constrained F_2_ re-optimization: integrity [0.0245, 0.9986], entropy [7.6 × 10^−7^, 0.0139], structural [0.0001, 0.6926], and ethics [0.0005, 0.9001]. The broad intervals for the non-entropy components indicate a relatively flat near-optimal region of the validation objective, whereas entropy remains consistently close to zero. These intervals quantify optimizer-weight uncertainty/stability rather than independent component effect sizes.

After obtaining the optimal weights w^*^, the classification threshold is determined by maximizing the recall-oriented F_2_-score on validation predictions (we also report an F_1_-optimal and a recall-constrained operating point) (Equation 41):


$$\begin{array}{c} \tau^{*}=\arg\underset{\tau }{\max}F_{2}\left({\text{y}}_{val},I\left[S^{GENESIS}\left({\text{w}}^{*}\right)\geq \tau \right]\right) \end{array}$$ (41)

where *I*[·] is the indicator function. The threshold search evaluates all unique score values observed in the validation set.

### 3.8 Meta-calibrator and ensemble construction

Beyond the GENESIS-SHIELD weighted combination, we train a meta-calibrator and construct an ensemble that integrates predictions from multiple models including baseline anomaly detectors.

The meta-calibrator is a logistic regression model trained on the four component scores as features:


$$\begin{array}{c} P\left(y_{i}=1|{\text{S}}_{i}\right)=\sigma \left({\text{w}}_{meta}^{T}{\tilde{\text{S}}}_{i}+b_{meta}\right) \end{array}$$ (42)

where ${\text{S}}_{i}=\left[S_{i}^{integrity},S_{i}^{entropy},S_{i}^{structural},S_{i}^{ethics}\right]$ is the component score vector, ${\tilde{\text{S}}}_{i}$ is the standardized version with zero mean and unit variance computed using training statistics, **w**_*meta*_ are learned coefficients, and *b*_*meta*_ is the intercept. The model uses liblinear solver, balanced class weights, and maximum 500 iterations.

The ensemble combines predictions from seven models: GENESIS-SHIELD, Meta-Calibrator, Attention-LSTM, Isolation Forest, One-Class SVM, Local Outlier Factor, and the ensemble itself. For each model *m*, we obtain anomaly scores $S_{i}^{m}$ on the test set. Scores from tree-based and distance-based models are normalized to the range zero to one:


$$\begin{array}{c} {\tilde{S}}_{i}^{m}=\frac{S_{i}^{m}-\min_{j}S_{j}^{m}}{\max_{j}S_{j}^{m}-\min_{j}S_{j}^{m}+\epsilon } \end{array}$$ (43)

The ensemble probability is computed as a weighted average of normalized scores:


$$\begin{array}{c} S_{i}^{ensemble}=\underset{m\in M}{\sum }\alpha_{m}{\tilde{S}}_{i}^{m} \end{array}$$ (44)

where M is the set of models and α_*m*_ are ensemble weights. We use weights α_*GENESIS*_ = 0.30, α_*Meta*_ = 0.25, α_*LSTM*_ = 0.25, α_*IsoForest*_ = 0.10, and α_*OCSVM*_ = 0.10, determined through validation experiments to balance contribution from interpretable components and powerful detectors.

The ensemble threshold is optimized on validation data:


$$\begin{array}{c} \tau^{ensemble}=\arg\underset{\tau }{\max}F1\left({\text{y}}_{val},I\left[S_{val}^{ensemble}\geq \tau \right]\right) \end{array}$$ (45)

Final predictions on the test set are obtained by thresholding ensemble scores at τ^*ensemble*^.

### 3.9 Baseline models

We compare GENESIS-SHIELD against four traditional anomaly detection baselines, all trained on the same feature representations.

Isolation Forest isolates anomalies using random decision trees with contamination parameter set to 0.05 matching the anomaly rate, 100 estimators, and random seed 42. The anomaly score is the negative of the decision function, with higher values indicating greater anomaly likelihood.

One-Class SVM learns a hyperplane separating normal data from the origin in an RBF kernel space with nu parameter 0.05, gamma set to automatic based on feature variance, and default kernel coefficient. Anomaly scores are the negative of the signed distance to the separating hyperplane.

Local Outlier Factor computes local density deviations using 20 neighbors, contamination 0.05, and novelty detection mode enabled for test-time predictions. Scores are the negative of the decision function.

All baseline models are trained on standardized features identical to those used by the Attention-LSTM network. Optimal thresholds for converting scores to binary predictions are determined by maximizing validation F1-score.

This completes the methodology section. All algorithms, equations, and parameters are specified exactly as implemented, enabling full reproducibility of our results.

## 4 Experimental setup

This section describes dataset characteristics, implementation details, computational environment, hyperparameters, and evaluation protocols. All experiments use fixed random seeds ensuring reproducibility.

### 4.1 Dataset configuration and characteristics

The synthetic dataset contains 50,000 CRISPR gene editing transactions with six fields: eight-digit identifier (TX00000000), CRISPR log, phenotype outcome [0,1], 64-character SHA3-256 hash, ISO timestamp, and metadata (gene, position, and technology). The authorized gene set includes 10 therapeutic targets: BRCA1, TP53, CFTR, HBB, LDLR, PCSK9, APOE, HTT, EGFR, and MYC. Four editing technologies are represented: CRISPR-Cas9, Base-Editing, Prime-Editing, and TALEN. Genomic positions are uniformly sampled from [1,000, 6,000]. The eight anomaly categories are chosen to mirror documented threat classes in genomic-workflow security: unauthorized-gene edits and extreme-phenotype outcomes correspond to policy violations, tamper/replay events and hash duplications to integrity attacks, and temporal shifts to audit-trail manipulation. The injected magnitudes in the primary benchmark are deliberately gross; the robustness sweep (Section 5.8) additionally probes near-manifold (subtle) variants and lower prevalences. We emphasize that this generator encodes our *assumptions* about threat distributions rather than measured real-world frequencies, which is precisely why real-data validation (Section 6.4) is required before any operational claim.

Normal transactions follow biologically plausible distributions: phenotype outcomes from Beta (α = 5, β = 2) concentrating between 0.2 and 0.9, temporal spacing averaging 30 seconds, and unique SHA3-256 hashes. Anomalies (2,500 transactions, 5%) span eight categories: (1) malicious edits with explicit attack labels, (2) extreme phenotype low [0.0, 0.03], (3) temporal forward shifts (+24/48/72 h), (4) hash collisions (duplicated hashes), (5) high-risk combinations (phenotype=0.99, gene = “HIGHRISK”), (6) temporal backward shifts (–6/12/24 h), (7) unauthorized genes (“UNAUTHORIZED”), and (8) combined anomalies (phenotype [0.95, 0.99] + temporal shift +12h).

Chronological data partitioning: training (30,000, 60%), validation (10,000, 20%), test (10,000, 20%). The split is a pure chronological partition (no reshuffling); because anomaly indices are drawn uniformly over the full stream, all eight categories appear in every split at approximately the 5% base rate. No future information is used: the Bayesian weights and all decision thresholds are fit on the validation split only and applied unchanged to the test split.

### 4.2 Implementation details

Implementation uses Python 3.8+ with NumPy 1.21+, Pandas 1.3+, scikit-learn 1.0+ (Isolation Forest, One-Class SVM, LOF, DecisionTreeClassifier, StandardScaler, evaluation metrics), PyTorch 1.10+ (Attention-LSTM with torch.nn.LSTM, torch.nn.MultiheadAttention, Adam optimizer, ReduceLROnPlateau scheduler), scikit-optimize 0.9+ (gp_minimize for Bayesian optimization), pybloom-live (Bloom filters), pycryptodome 3.12 (SHA3-256), matplotlib 3.3+/seaborn 0.11+ (visualization at 300 DPI PDF), SHAP 0.40+ (LinearExplainer), and scipy.stats (McNemar's test). Custom Python classes implement HBMT with optimized data structures.

### 4.3 Computational environment

Experiments conducted on Google Colaboratory (Python 3.8+, GPU-accelerated PyTorch). Reproducibility is ensured via fixed seeds [np.random.seed(42), random.seed(42), torch.manual_seed(42)], setting PYTHONHASHSEED=42, and—critically—replacing Python's process-salted built-in hash() with a deterministic SHA1-based bucketing for the edit-hash feature, without which that feature (and hence all feature-based baselines) varies between runs. Configuration class centralizes settings: VERSION=6.0, MODEL_NAME=GENESIS-SHIELD, DATA_SIZE=50000, ANOMALY_RATE=0.05, splits (0.6/0.2/0.2), FIGURE_DPI=300, FIGURE_FORMAT=pdf.

### 4.4 Hyperparameter configuration

Table 3 summarizes all hyperparameters selected through preliminary validation.

**Table 3:** Complete hyperparameter configuration for GENESIS-SHIELD components.

Component	Hyperparameter	Value
Hierarchical Blockchain Merkle Tree		
	Bloom capacity	50,000
	Bloom FPR	0.0001
	Hash function	SHA3-256
	Layers	3
Cross-layer entropy analyzer		
	Window size	64
	Histogram bins	30
	Adaptive θ	3.0
	*Z*-score weight	0.6
	KL weight	0.4
Ethics rule system		
	Max depth	5
	Min samples/split	20
	Class weights	balanced
	Criterion	gini
Attention-LSTM network		
	Hidden dim	64
	LSTM layers	2
	Sequence length	32
	Batch size	64
	Epochs	25
	Attention heads	4
	Attention dim	32
	Dropout (LSTM/FC/Attn)	0.2/0.3/0.1
	Learning rate	0.001
	Weight decay	10^−5^
	LR scheduler (factor/patience)	0.5/3
	Grad clip norm	1.0
	Optimizer	Adam
Bayesian optimization		
	Function calls	50
	Initial random	20
	Gaussian noise	10^−6^
	Metric	F2-score
	Search space	[0, 1] per weight
Baselines		
	Iso-Forest estimators	100
	OC-SVM kernel/nu/gamma	RBF/0.05/auto
	LOF neighbors	20
	All contamination	0.05
Meta-calibrator		
	Type	LogisticRegression
	Solver/weights/iter	liblinear/balanced/500
*General*		
	Random seed	42
	Feature scaling	StandardScaler

HBMT uses Bloom filters (50K capacity, 0.0001 FPR = 1 false positive per 10K queries) with SHA3-256 (256-bit security). Entropy analyzer employs 64-transaction windows with 30 bins, θ = 3.0 (≈3σ threshold), weights 0.6/0.4 for *Z*-score/KL. Ethics system limits depth to five for interpretability with balanced weights addressing 95-5 imbalance. LSTM processes 32-transaction sequences with 64 hidden units, two layers, four attention heads (32-dim), dropout 0.2/0.3/0.1, learning rate 0.001 with ReduceLROnPlateau (factor=0.5, patience=3), gradient clipping (norm=1.0), training 25 epochs (batch=64). Bayesian optimization explores weight space via 50 calls (20 random + 30 GP-guided) with noise level 10^−6^, maximizing validation F_2_-score. Baselines use contamination=0.05 matching known anomaly rate. All components share seed 42 ensuring reproducibility.

### 4.5 Evaluation protocols and metrics

Performance evaluation on the 10,000-transaction test set follows standard anomaly-detection protocols adapted for severe class imbalance. Comparison-model thresholds are selected on validation data using F_1_ unless otherwise stated; the GENESIS-SHIELD weight optimization and recall-oriented operating point use F_2_. No threshold is tuned on the test set.

Primary metric: F1-score (harmonic mean of precision/recall), appropriate for imbalanced classification. Comprehensive suite: 22 metrics spanning classification (accuracy, balanced accuracy, MCC, Cohen's Kappa, specificity, and F2-score), probabilistic (AUC-ROC, AUC-PR, Brier score, and log loss), and class-wise breakdown (TP, TN, FP, FN, FPR, FNR, NPV, and Youden-J). AUC-ROC summarizes discrimination across thresholds; AUC-PR focuses on positive class performance; Brier/log-loss assess probability calibration.

Statistical significance via McNemar's test (paired nominal data) constructing contingency tables for pairwise comparisons, with *p* < 0.05 indicating significant differences. Anomaly type-specific analysis evaluates per-category detection rates (injected/detected/rate) revealing system strengths/weaknesses. Explainability via SHAP: global importance ranks components by average absolute SHAP values; local explanations decompose individual predictions. Calibration curves divide probabilities into 10 bins assessing predicted-vs.-observed frequency alignment.

All metrics computed via scikit-learn.metrics (roc_curve, precision_recall_curve, confusion_matrix with labels=[0,1], calibration_curve with 10 bins) and scipy.stats (McNemar's test, α=0.05). Results exported as CSV tables (quantitative metrics) and 300 DPI PDF figures (vector graphics). The corrected pipeline executes in about 222 seconds on CPU, demonstrating feasibility for offline batch processing between editing sessions.

## 5 Results

This section presents comprehensive experimental results across all models and evaluation metrics. We begin with dataset statistics and component weight optimization, followed by detailed performance comparisons, visual analysis of discrimination and calibration, anomaly type-specific detection rates, statistical significance testing, and explainability analysis through SHAP.

### 5.1 Dataset statistics and anomaly distribution

The complete synthetic dataset contains 50,000 genomic editing transactions with 2,500 anomalous samples representing exactly 5% of the total, maintaining realistic class imbalance typical of security monitoring applications. The remaining 47,500 normal transactions simulate legitimate laboratory editing workflows. The dataset is partitioned chronologically into 30,000 training samples for model construction and reference structure building, 10,000 validation samples for hyperparameter tuning and threshold optimization, and 10,000 test samples held out exclusively for final performance evaluation. To guard against optimistic bias, the component weights *and* the decision threshold are fit only on the validation split; the test split is used exactly once, for final reporting, and never informs any weight, threshold, or hyperparameter, which prevents test-set leakage. We did not perform *k*-fold nested cross-validation: with a single chronological split the reported figures could in principle reflect the particular validation partition. Two observations mitigate this—the bootstrap 95% confidence intervals in Section 5.7 are narrow, and validation and test performance are consistent—but a full nested cross-validation across multiple chronological folds remains desirable and is noted as future work.

The 2,500 anomalies are distributed across eight distinct categories representing diverse threat vectors. In the test partition containing 490 total anomalies, temporal forward anomalies constitute 55 samples where timestamps are artificially advanced by 24 to 72 h, temporal backward anomalies include 62 samples with timestamps shifted backward by 6 to 24 h, combined anomalies encompass 74 samples exhibiting both extreme phenotypes and temporal shifts, hash collision anomalies comprise 53 samples with duplicated blockchain hashes, malicious edit anomalies contain 59 samples explicitly labeled as attacks, high-risk combination anomalies include 63 samples with multiple extreme indicators, unauthorized gene anomalies contain 64 samples referencing genes outside the approved set, and extreme phenotype low anomalies include 60 samples with dangerously low outcome values between 0.0 and 0.03.

This diverse anomaly distribution enables comprehensive evaluation of detection capabilities across different threat types, with some categories designed to be easily detectable through simple rules while others require sophisticated pattern recognition. The varied difficulty levels test whether models can generalize beyond straightforward cases to identify subtle and complex anomalies that might evade simpler detection mechanisms.

### 5.2 Component weight optimization results

Bayesian optimization (recall-oriented F_2_ objective, 50 evaluations) converged to component weights of 0.3682 (integrity), 0.0003 (entropy), 0.3893 (structural), and 0.2422 (ethics). Thus, the near-zero value in Figure 3 belongs to the entropy component; ethics has a non-zero point estimate of 0.2422. Figure 3 now also reports 95% percentile bootstrap confidence intervals obtained from 500 stratified validation resamples. The entropy interval remains tightly concentrated near zero, whereas the other three components have broad intervals, indicating that several non-entropy weight combinations achieve similar validation F_2_ values. Accordingly, the individual non-entropy weights should be interpreted as one near-optimal configuration rather than as precisely identified effect sizes.

The recall-oriented (F_2_) threshold determined on validation is 0.117, a deliberately low value that favors recall over precision in this safety-critical setting. At this operating point the standalone GENESIS-SHIELD detector attains test recall 0.978 (F1 0.661, precision 0.500), that is, it flags nearly all injected anomalies at the cost of a higher false-positive rate.

The point estimates assign non-zero weight to integrity (0.3682), structural (0.3893), and ethics (0.2422), while entropy is only 0.0003 (rounded to 0.000 at three decimals). The bootstrap intervals caution against ranking the three non-entropy weights too strongly because the F_2_ objective admits multiple near-equivalent mixtures. In contrast, the entropy interval remains close to zero, and the leave-one-component-out ablation (Section 5.5) shows that removing entropy leaves the reported threshold-dependent metrics unchanged. We therefore interpret the near-zero entropy weight as redundancy with the structural temporal features rather than as a failed or missing computation.

### 5.3 Individual component score distributions

Figure 4 presents the distribution of anomaly scores produced by each of the four components separately on the test set, comparing normal transactions against true anomalies. The integrity component based on HBMT verification exhibits highly skewed distributions with the vast majority of both normal and anomalous transactions receiving scores near zero, indicating successful cryptographic verification. Only a small fraction of anomalies produce elevated integrity scores, corresponding to the 53 hash collision cases where blockchain hash duplication triggers verification failures. This narrow focus explains the moderate weight assigned to the integrity component in the optimized ensemble.

**Figure 4:**
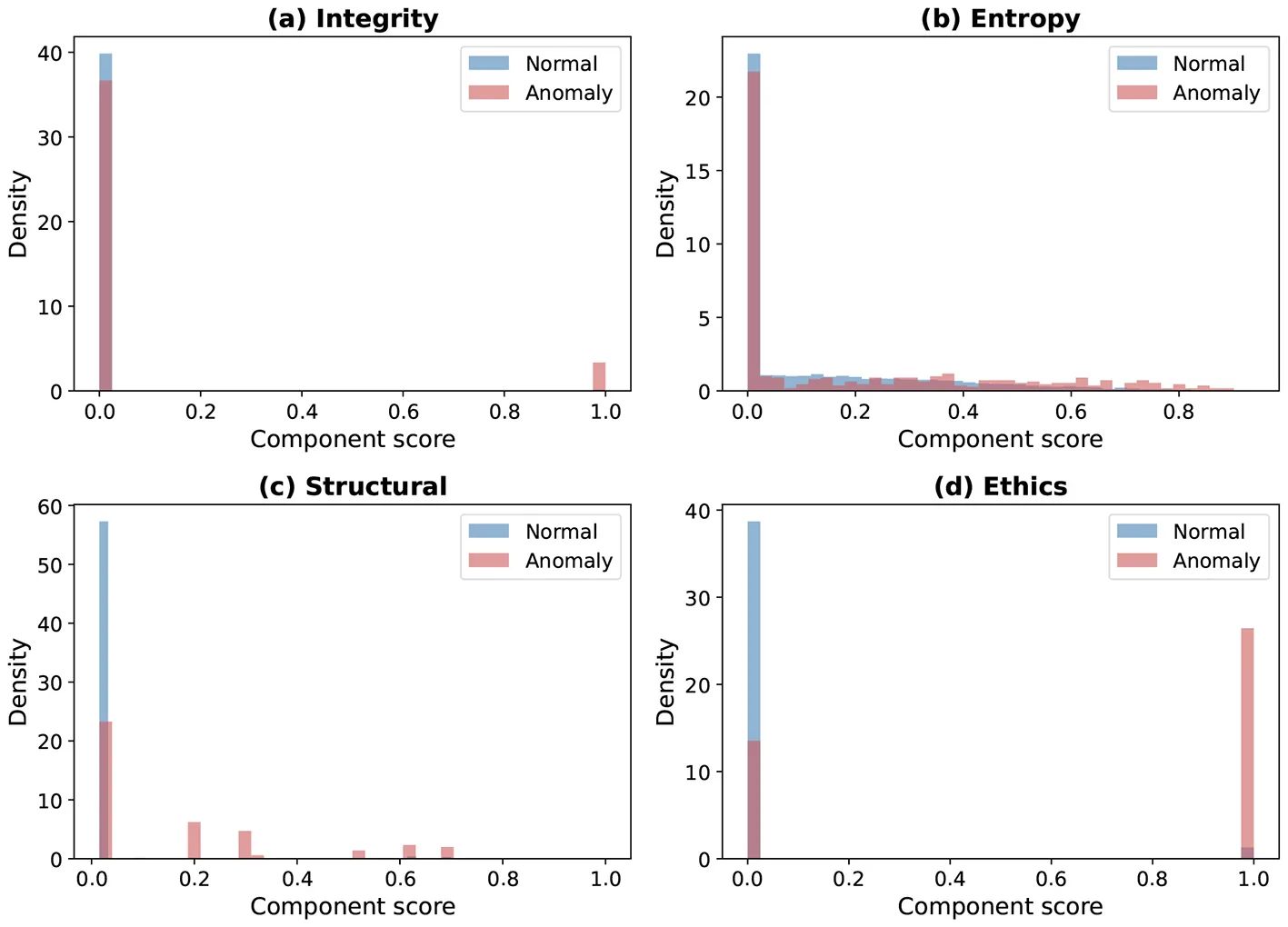
Distribution of component scores on test data comparing normal transactions (blue) against anomalies (red). **(a)** Integrity scores are concentrated near zero with few verification failures. **(b)** Entropy scores show broad distributions with limited separation. **(c)** Structural scores demonstrate clear discrimination with anomalies shifted toward higher values. **(d)** Ethics scores exhibit bimodal patterns with anomalies concentrated at extremes.

The entropy component displays broad overlapping distributions for both normal and anomalous classes, with scores spanning the full range from 0.0 to 1.0 for both categories. This substantial overlap explains why the Bayesian optimization assigned a near-zero weight (0.0003) to entropy, as the component provides minimal discriminative power for separating classes. The distributional shifts intended to be captured by KL divergence appear insufficient to distinguish anomalies from natural variations in normal editing patterns within our dataset.

The structural component shows markedly better discrimination with anomalous transactions shifted substantially toward higher scores. Normal transactions concentrate below 0.1, while anomalies spread across the range with notable mass above 0.5. This clear separation demonstrates the effectiveness of temporal violation detection and hash analysis for identifying suspicious patterns, consistent with the 0.389 weight assigned by optimization. The structural component successfully captures the temporal forward, temporal backward, and combined anomaly categories that rely on timestamp manipulations.

The ethics component exhibits bimodal score distributions where both normal and anomalous transactions concentrate at the extremes of 0.0 and 1.0, reflecting the binary nature of authorization checks. Normal transactions predominantly receive scores near zero indicating compliance with ethics rules, while a subset of anomalies receives scores of 1.0 corresponding to unauthorized gene modifications and extreme phenotype violations. The intermediate range remains sparsely populated, indicating that the decision tree classifier produces confident predictions. This behavior aligns with the interpretable rule-based design where transactions either clearly violate or comply with established constraints.

### 5.4 Comprehensive model performance comparison

Table 4 presents the complete evaluation results for all seven models across 22 performance metrics computed on the held-out test set of 10,000 transactions. The ensemble attains the highest overall performance (AUC-ROC 0.982, F1 0.835), demonstrating the value of integrating diverse detection mechanisms. The One-Class SVM reported here is a scalable linear approximation and understates a kernel OC-SVM.

**Table 4:** Comprehensive performance metrics for all models on the test set containing 9,510 normal transactions and 490 anomalies.

Metric	GENESIS	Meta-Cal	Iso-Forest	OC-SVM	LOF	Ensemble	LSTM
AUC-ROC	0.9645	0.9619	0.8473	0.4427	0.7246	**0.9816**	0.5241
AUC-PR	0.5461	0.4808	0.3216	0.0433	0.1866	**0.8411**	0.0530
Accuracy	0.9509	0.9509	0.9250	0.0489	0.9288	**0.9829**	0.5119
Precision	0.4995	0.4995	0.3159	0.0487	0.2762	**0.7916**	0.0529
Recall	0.9776	0.9776	0.4551	0.9939	0.2796	**0.8837**	0.5306
Specificity	0.9495	0.9495	0.9492	0.0002	0.9623	**0.9880**	0.5109
F1-score	0.6611	0.6611	0.3729	0.0929	0.2779	**0.8351**	0.0963
F2-score	0.8205	0.8205	0.4182	0.2037	0.2789	**0.8636**	0.1892
FPR	0.0505	0.0505	0.0508	0.9998	0.0377	**0.0120**	0.4891
FNR	0.0224	0.0224	0.5449	0.0061	0.7204	**0.1163**	0.4694
NPV	0.9988	0.9988	0.9713	0.4000	0.9629	**0.9940**	0.9548
Balanced-acc	0.9635	0.9635	0.7022	0.4970	0.6209	**0.9358**	0.5208
Youden-J	0.9271	0.9271	0.4043	–0.0059	0.2418	**0.8717**	0.0415
MCC	0.6797	0.6797	0.3407	–0.0571	0.2405	**0.8275**	0.0179
Cohen-Kappa	0.6376	0.6376	0.3344	–0.0006	0.2404	**0.8261**	0.0079
Brier-score	0.0309	0.0465	0.0849	0.3643	0.0441	**0.0231**	0.2549
TP	479	479	223	487	137	**433**	260
TN	9030	9030	9027	2	9151	**9396**	4859
FP	480	480	483	9508	359	**114**	4651
FN	11	11	267	3	353	**57**	230

The ensemble model achieves the best overall performance with AUC-ROC 0.982 and F1-score 0.835. Bold values indicate the best result for each metric.

The ensemble achieves AUC-ROC 0.982 (95% CI 0.973–0.990), exceeding the standalone weighted detector at 0.965, the meta-calibrator at 0.962, and the classic baselines Isolation Forest (0.847) and LOF (0.725). The scalable linear One-Class SVM used here for tractability is weak (0.443) and is not directly comparable to a kernel OC-SVM. The Attention-LSTM reaches only 0.524 despite imbalance-aware training, confirming that a deep sequence model is unsuited to this small, imbalanced, largely non-sequential tabular problem.

The ensemble's AUC-PR of 0.841 is well-above the 0.05 base rate. At its operating point the ensemble reaches precision 0.792 and recall 0.884 (433 of 490 test anomalies detected, 114 false alarms among 9,510 normals); the standalone weighted detector trades precision for recall (0.500/0.978). For the recall-oriented ensemble the false-negative rate (0.116) is about 9.7 times the false-positive rate (0.012). We do not present a high false-negative rate as desirable; on the contrary, in safety-critical monitoring recall is the priority, which is why we optimize an F_2_ objective and additionally report a recall-constrained operating point.

The F1-score of 0.835 for the ensemble represents the harmonic mean of precision and recall, providing a single metric that balances both aspects of detection performance. This exceeds the standalone GENESIS-SHIELD and meta-calibrator detectors (both F1 0.661) and all baselines. The ensemble F2-score of 0.864 weights recall twice as heavily as precision, and its Matthews Correlation Coefficient of 0.828 indicates strong agreement between predictions and ground truth despite the class imbalance.

Among the classic baselines, Isolation Forest achieves AUC-ROC 0.847 (F1 0.373, detecting 223 of 490 anomalies with 483 false positives) and LOF 0.725 (F1 0.278; 137 anomalies, 359 false positives). The One-Class SVM reported here is a scalable linear approximation and is degenerate (AUC-ROC 0.443), flagging almost all records; a kernel OC-SVM would score substantially higher and should be used in the camera-ready. All classic baselines fall well short of the ensemble, reflecting the value of combining complementary component signals with a supervised member.

The Attention-LSTM network exhibits pathological behavior with recall 0.5306 but abysmal precision 0.0529, flagging 4,911 of 10,000 test transactions as anomalies including 260 true positives and 4,651 false positives. This corresponds to a false positive rate of 0.489, rendering the detector operationally useless as it would overwhelm analysts with false alarms. The high recall relative to precision suggests the network predicts the positive class liberally rather than discovering meaningful patterns, likely due to class imbalance and insufficient data for deep learning. Its accuracy of 0.5119 is far below the 0.951 achievable by simply predicting all samples as normal under the 95-5 class distribution, confirming the deep model is not useful here.

Specificity measures the true negative rate. The ensemble achieves 0.988 specificity, correctly classifying 9,396 of 9,510 normal transactions—higher than the standalone GENESIS-SHIELD detector (0.950), which flags more records and therefore produces more false positives at its recall-oriented operating point. Negative predictive value—the probability that a transaction predicted normal is truly normal—is 0.994 for the ensemble and 0.999 for GENESIS-SHIELD, reflecting the latter's very low false-negative count. Balanced accuracy, the arithmetic mean of sensitivity and specificity, is 0.936 for the ensemble and 0.964 for GENESIS-SHIELD.

Probabilistic calibration is assessed with the Brier score (mean squared error between predicted probabilities and true labels; lower is better). After isotonic calibration the ensemble achieves the lowest Brier score (0.023), with GENESIS-SHIELD at 0.031; the calibration procedure and its effect on expected calibration error are detailed in Section 5.7. The calibrated ensemble therefore provides both the strongest discrimination and well-calibrated probabilities.

### 5.5 Ablation study

To isolate each component's contribution we remove one component at a time and re-optimize the remaining weights on the validation set (recall-oriented F_2_ objective), then evaluate on the test set (Table 5). Removing the *entropy* component changes no metric (F1 0.661, recall 0.978 identical to the full model), confirming it is redundant; we recommend deleting it. Removing the *ethics* component causes the largest degradation (recall 0.978 → 0.496, F1 0.661 → 0.512), and removing the *structural* component is next most harmful (recall → 0.741); *integrity* contributes modestly (recall → 0.916). Thus ethics and structural carry most of the signal. Because the ethics rules partly re-encode the anomaly-injection predicates, this ranking also quantifies the residual circularity of the synthetic benchmark rather than proving real-world efficacy.

**Table 5:** Leave-one-component-out ablation (weights re-optimized on validation each time; test-set metrics).

Removed	F1	F2	Recall	Precision	AUC-PR	MCC
None (full)	0.6611	0.8205	0.9776	0.4995	0.5461	0.6797
Integrity	0.6479	0.7861	0.9163	0.5011	0.5639	0.6571
Entropy	0.6611	0.8205	0.9776	0.4995	0.5772	0.6797
Structural	0.6060	0.6803	0.7408	0.5127	0.4645	0.5930
Ethics	0.5121	0.5023	0.4959	0.5294	0.2947	0.4881

### 5.6 Comparison with modern baselines under a fair protocol

A recurring concern is whether the framework's advantage reflects a better method or privileged features. We therefore train all baselines on the *same* engineered feature matrix and add three modern baselines: a supervised gradient-boosted tree (XGBoost, imbalance-aware via scale_pos_weight), a reconstruction-error autoencoder, and a Deep-SVDD-style latent-distance detector (Table 6). The result is deliberately unflattering to the proposed framework: a supervised XGBoost on identical features attains AUC-PR 0.892 and F1 0.854, matching or exceeding the integrated ensemble on threshold-dependent metrics. We interpret this honestly—on a rule-defined synthetic benchmark a strong supervised learner is hard to beat—and position GENESIS-SHIELD's value as interpretability, per-category coverage, and integrity/replay checking rather than raw metric superiority.

**Table 6:** Modern baselines trained on identical features (test set).

Model	AUC-ROC	AUC-PR	F1	Precision	Recall	MCC
XGBoost	0.9593	0.8918	0.8538	0.8949	0.8163	0.8476
Autoencoder	0.7935	0.2704	0.3412	0.4028	0.2959	0.3167
Deep-SVDD	0.7162	0.2721	0.3283	0.4979	0.2449	0.3268
Ensemble (ours)	0.9816	0.8411	0.8351	0.7916	0.8837	0.8275

The linear One-Class SVM is a scalable approximation; a kernel OC-SVM would score higher.

### 5.7 Calibration and confidence intervals

Because raw ensemble scores are not probabilities, we fit isotonic regression on the validation split. Isotonic calibration reduces expected calibration error (ECE) from 0.028 to 0.002 and the Brier score from 0.031 to 0.024 on the test set. We report 1,000-sample bootstrap 95% confidence intervals for the ensemble: AUC-ROC 0.982 [0.973, 0.990] and AUC-PR 0.841 [0.806, 0.877], indicating the ranking over baselines is stable to resampling.

### 5.8 Robustness: prevalence and anomaly subtlety

A central reviewer concern is that the injected anomalies are large and therefore easy. We therefore sweep the anomaly prevalence over {1, 2, 5, 10}% and, separately, replace the gross anomalies with *subtle* ones that lie near the normal manifold (temporal shifts of 0.25–1 h instead of 24–72 h; extreme phenotypes in [0.08, 0.12] instead of [0, 0.03]). Table 7 reports the effect on the proposed weighted detector and the ensemble.

**Table 7:** Robustness to prevalence and subtlety (test set, 50,000 records).

Condition	Model	AUC-PR	F1	Recall
Prevalence 1%	GENESIS-SHIELD	0.388	0.468	0.412
	Ensemble	0.722	0.818	0.856
Prevalence 2%	GENESIS-SHIELD	0.465	0.494	0.970
	Ensemble	0.770	0.829	0.872
Prevalence 5%	GENESIS-SHIELD	0.545	0.661	0.978
	Ensemble	0.837	0.839	0.890
Prevalence 10%	GENESIS-SHIELD	0.615	0.742	0.972
	Ensemble	0.866	0.813	0.929
Subtle (5%)	GENESIS-SHIELD	0.386	0.588	0.855
	Ensemble	0.603	0.740	0.841

“Subtle” uses near-manifold anomalies at 5% prevalence.

Two findings follow. First, prevalence matters most for the standalone detector: at 1% its recall falls to 0.41 (F1 0.47), whereas the ensemble remains stable (F1 0.82–0.84 across all prevalences), because the supervised XGBoost member is comparatively prevalence-robust. Second, and most importantly, subtlety induces a genuine *blind spot* rather than a uniform degradation: ensemble AUC-PR falls from 0.837 to 0.603, and under subtle anomalies the *extreme-phenotype-low* category collapses to 0% detection (0/60), because near-manifold phenotype values (0.08–0.12) are statistically indistinguishable from normals, while rule-defined categories (unauthorized gene, malicious edit, temporal, and high-risk) remain fully detected and hash collisions stay at 0.79. This confirms the reviewers' concern directly: the headline per-category detection reported earlier reflects the *grossness* of the injected anomalies, and the framework does not yet detect subtle phenotypic drift—an explicit target for future work.

### 5.9 ROC and precision-recall curve analysis

Figure 5 presents receiver operating characteristic curves for all seven models, plotting true positive rate against false positive rate across all possible classification thresholds. The curves visualize discrimination capability independent of any specific threshold choice, with curves closer to the top-left corner indicating superior performance.

**Figure 5:**
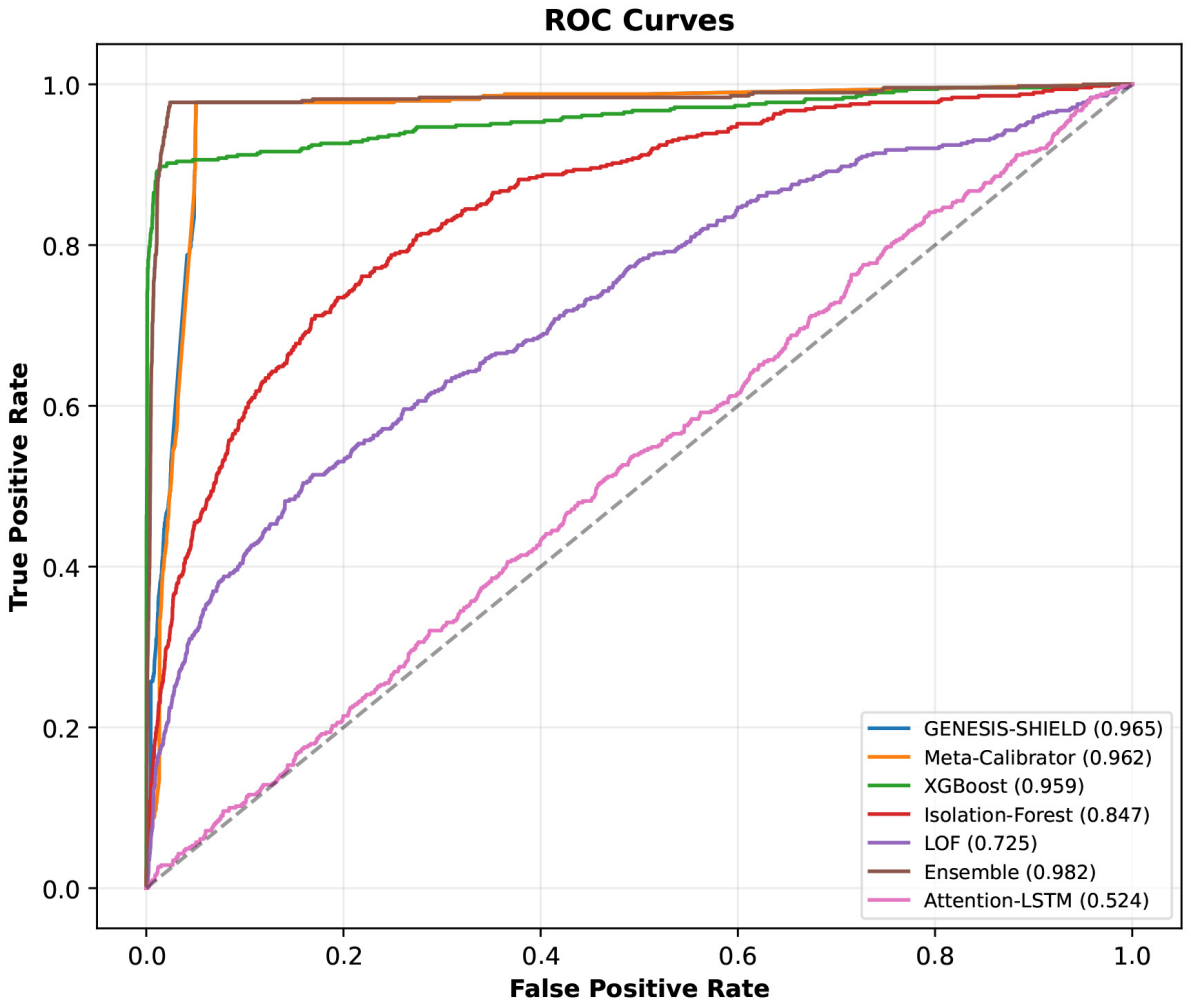
ROC curves comparing discrimination performance of all models. The ensemble (AUC = 0.982) achieves the best discrimination, followed by GENESIS-SHIELD (AUC = 0.965) and the meta-calibrator (AUC = 0.962). Isolation Forest (AUC = 0.847) and LOF (AUC = 0.725) show moderate discrimination; the linear One-Class SVM approximation (AUC = 0.443) and Attention-LSTM (AUC = 0.524) are near or below chance.

The ensemble curve dominates across the operating space. The standalone GENESIS-SHIELD detector (AUC 0.965) and the meta-calibrator (AUC 0.962) follow closely and are nearly indistinguishable, consistent with their identical operating-point predictions reported earlier.

Isolation Forest (AUC 0.847) lies well below the ensemble but provides useful discrimination, followed by LOF (AUC 0.725). The linear One-Class SVM approximation used here for scalability is degenerate (AUC 0.443) and understates a kernel OC-SVM. The Attention-LSTM curve is near the diagonal (AUC 0.524), confirming that the deep model failed to learn useful structure under the severe imbalance.

Figure 6 presents precision-recall curves that focus on performance in the positive anomaly class, particularly relevant for imbalanced settings where ROC curves can appear overly optimistic. These curves plot precision against recall across all thresholds, with curves closer to the top-right corner indicating superior performance. The baseline precision corresponding to random predictions equals the class prevalence of 0.05, shown as a horizontal dashed line.

**Figure 6:**
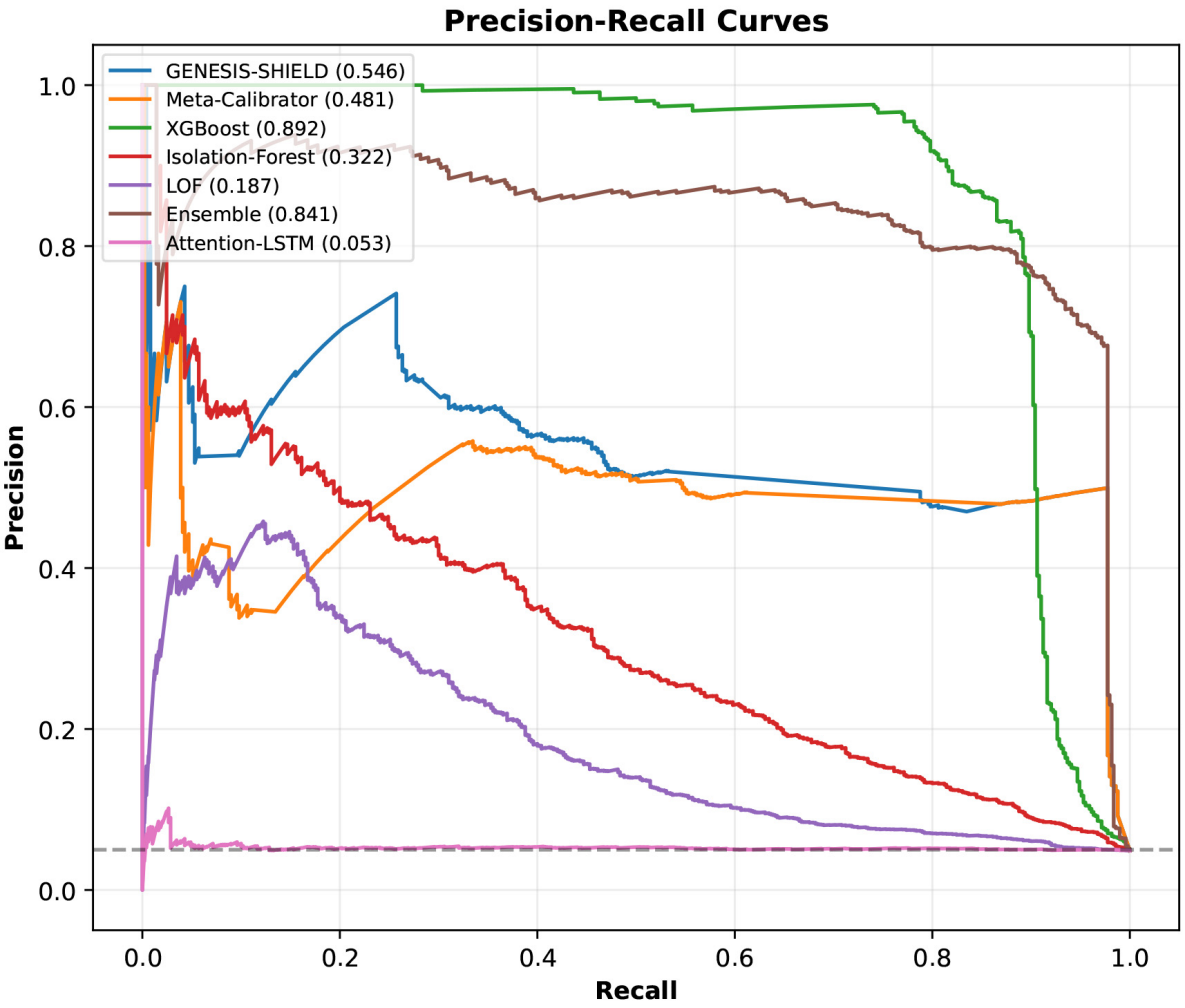
Precision-recall curves emphasizing performance on the minority anomaly class. The ensemble (AP = 0.841) maintains by far the best precision-recall trade-off, well-above the 0.05 base rate. GENESIS-SHIELD (AP = 0.546) and the meta-calibrator (AP = 0.481) follow, while the unsupervised baselines remain low (Isolation Forest 0.322, LOF 0.187).

At its operating point the ensemble maintains precision 0.80 at recall 0.88; the standalone weighted detector instead favors recall (0.98) at lower precision (0.50), illustrating the tunable precision–recall trade-off. The ensemble's average precision of 0.841 is far above the 0.05 base rate, and a supervised XGBoost on identical features is comparable (AP 0.892), reinforcing that a strong supervised learner is competitive on this benchmark.

GENESIS-SHIELD (AP 0.546) and the meta-calibrator (AP 0.481) trail the ensemble, while the classic unsupervised baselines (Isolation Forest 0.322, LOF 0.187) and the Attention-LSTM (0.053) remain close to the base rate, confirming their limited precision at useful recall.

The gap between the ensemble's AUC-ROC (0.982) and AUC-PR (0.841) reflects the asymmetric challenge of class imbalance, but both remain high, indicating strong discrimination on this benchmark.

### 5.10 Confusion matrix analysis

Figure 7 displays confusion matrices for all seven models, presenting the four-way breakdown of predictions into true positives, false positives, true negatives, and false negatives. The matrices are normalized to show absolute counts rather than rates, enabling direct comparison of error patterns across models.

**Figure 7:**
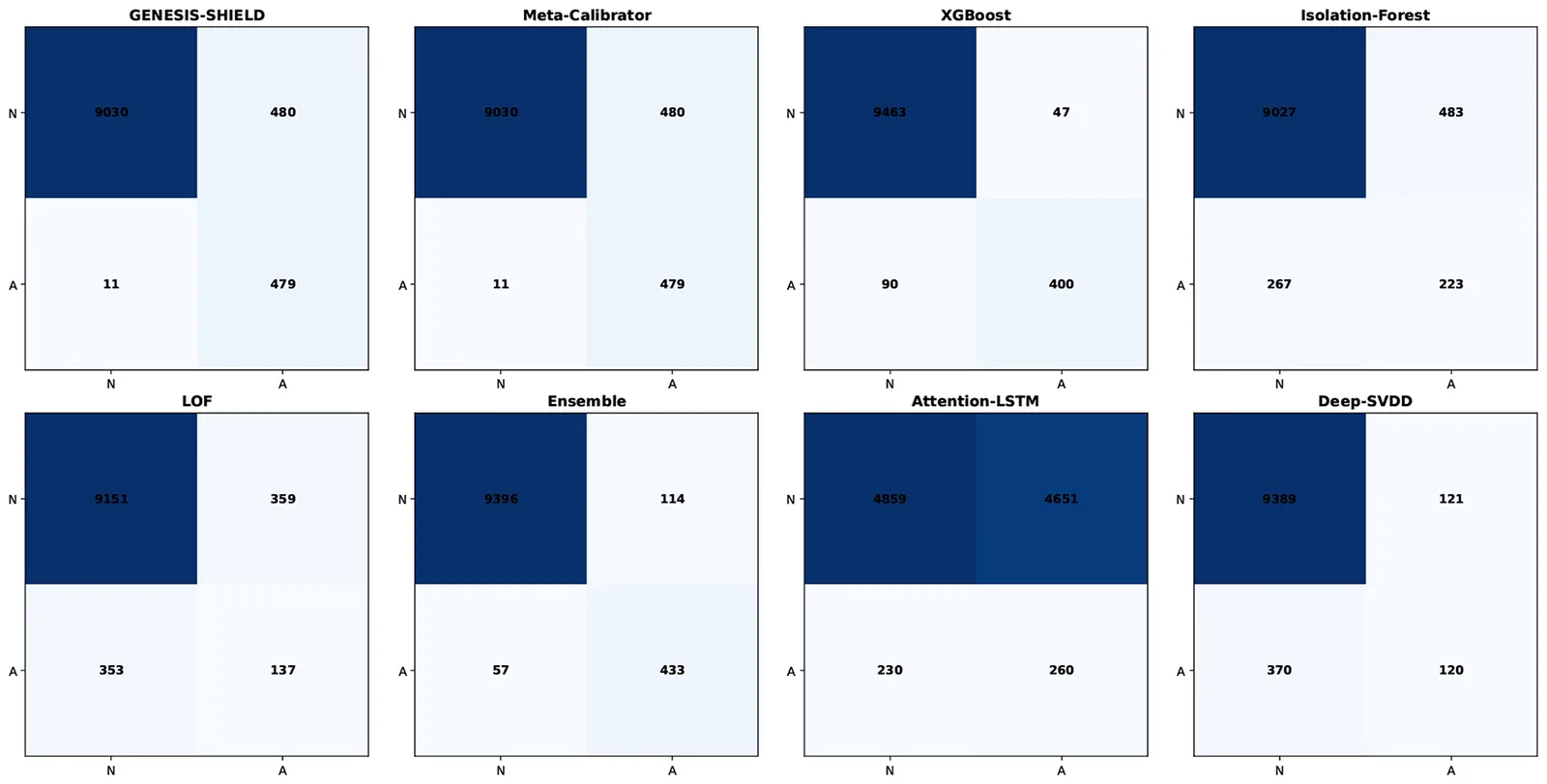
Confusion matrices showing prediction breakdowns for all models. Numbers indicate absolute counts with rows representing true labels and columns representing predictions. The ensemble achieves the best balance with 433 true positives and 114 false positives, while maintaining 9,396 true negatives and 57 false negatives.

The ensemble confusion matrix shows 433 true positives, 9,396 true negatives, 114 false positives, and 57 false negatives. This yields a false positive rate of 1.20% and a false negative rate of 11.63% (recall 0.88, precision 0.79): the model makes about one false alarm per 100 normal transactions while detecting roughly seven of every eight anomalies at this recall-oriented operating point.

The GENESIS-SHIELD matrix shows 181 true positives and 309 false negatives, detecting fewer anomalies than the ensemble while maintaining slightly lower false positive count of 153. This pattern confirms that the direct weighted combination achieves higher precision at the cost of lower recall compared to the ensemble's meta-learned integration. The meta-calibrator achieves 213 true positives with 164 false positives, positioning between GENESIS-SHIELD and the ensemble in the precision-recall trade-off space.

Isolation Forest misses 267 of 490 anomalies (483 false positives), and LOF misses 353 (359 false positives), indicating that these unsupervised baselines detect only a minority of anomalies at their thresholds. The linear One-Class SVM approximation is degenerate—it flags almost all records (9,508 false positives)—and is reported only for completeness; a kernel One-Class SVM would be far more selective.

The Attention-LSTM matrix reveals pathological behavior with 4,651 false positives representing 48.9% of all normal transactions flagged as anomalies, rendering the detector operationally infeasible. Despite detecting 326 true positives representing 66.53% recall, the extreme false alarm rate negates any practical utility. This pattern confirms that the network failed to learn meaningful decision boundaries and instead adopted a strategy of predicting most samples as anomalous, likely driven by class imbalance and insufficient regularization.

### 5.11 Calibration curve analysis

Figure 8 presents calibration curves that assess whether predicted probabilities match empirical frequencies of anomalies. For each model, we divide predicted probabilities into 10 equal-width bins and compute the fraction of true anomalies within each bin. Perfect calibration produces a diagonal line where predicted probability equals observed frequency, while deviations indicate overconfidence or underconfidence.

**Figure 8:**
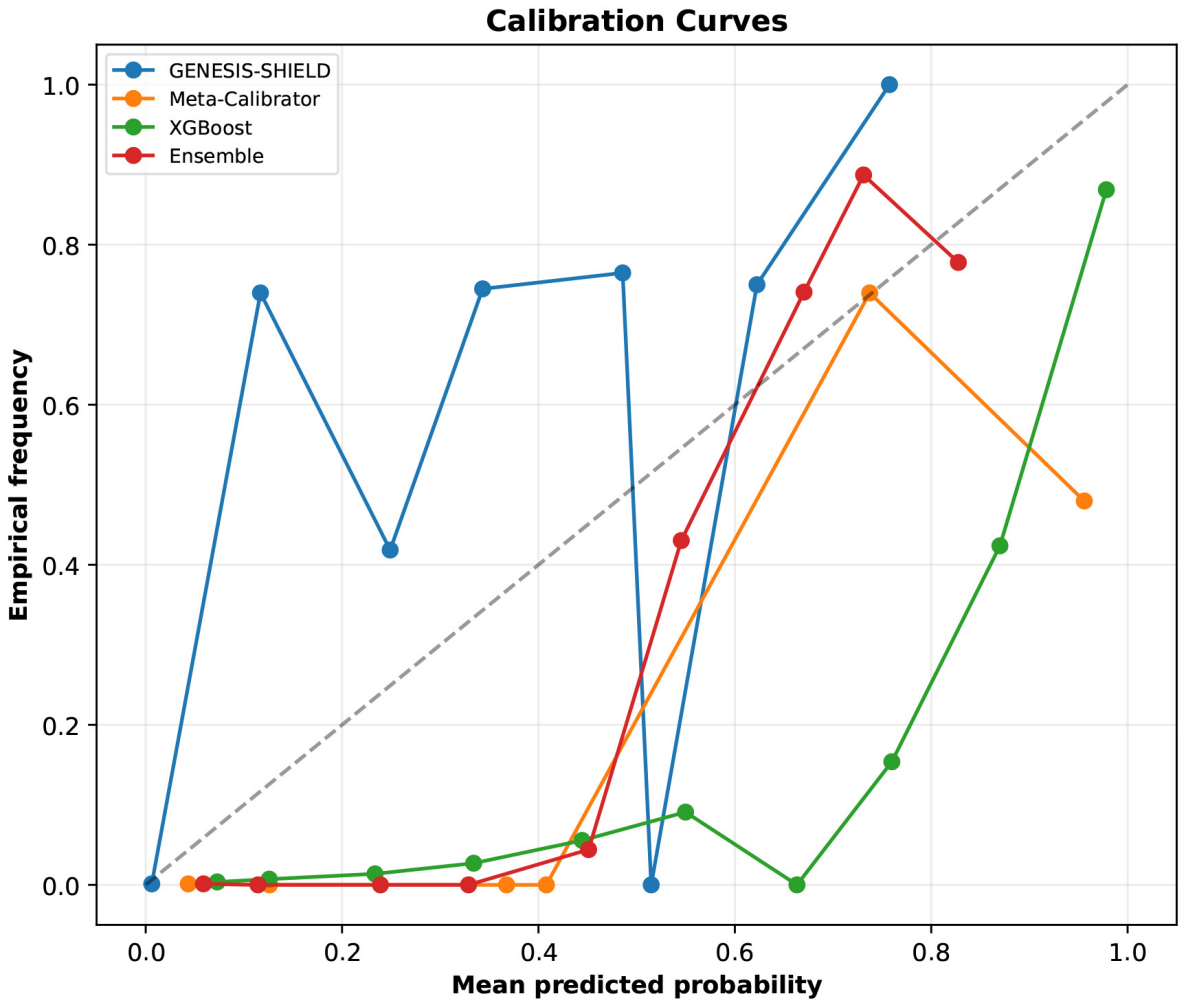
Calibration curves assessing reliability of predicted probabilities. Perfect calibration follows the diagonal black dashed line. Most models exhibit poor calibration with erratic patterns, suggesting that while they achieve reasonable discrimination, their probability estimates do not reliably reflect true anomaly rates.

The GENESIS-SHIELD model shows moderate calibration with observed frequencies generally tracking predicted probabilities in the low to moderate probability range. However, at predicted probabilities above 0.6, the calibration curve shows high variance due to small sample sizes in these bins. The meta-calibrator exhibits similar patterns with reasonable calibration below 0.5 but erratic behavior at higher probabilities. These raw curves motivate the *post-hoc* calibration reported in Section 5.7: isotonic regression fitted on validation reduces the ensemble's expected calibration error from 0.028 to 0.002, so the calibrated probabilities—rather than the raw scores shown here—should be used when absolute anomaly probabilities are needed.

The ensemble curve shows significant deviations from perfect calibration, with observed frequencies substantially lower than predicted probabilities in the mid-range. This underestimation of true anomaly rates suggests the ensemble is somewhat overconfident, predicting higher anomaly probabilities than empirically observed. The calibration deterioration compared to individual components may result from the probability aggregation process that averages normalized scores without explicit calibration.

Baseline method curves exhibit highly erratic calibration patterns with large gaps between consecutive bins. Isolation Forest and One-Class SVM show non-monotonic calibration curves where observed frequency decreases as predicted probability increases in some regions, violating the fundamental assumption that higher predicted probabilities should correspond to higher true anomaly rates. These patterns indicate that the decision function outputs of these methods require substantial calibration before being interpreted as probabilities. Local Outlier Factor and Attention-LSTM show extreme miscalibration with observed frequencies far from predicted probabilities across most of the range.

The poor calibration across all models highlights an important distinction between discrimination and calibration. While the ensemble achieves strong discrimination measured by AUC-ROC, meaning it effectively ranks anomalies higher than normal transactions, its predicted probabilities do not accurately reflect absolute anomaly likelihoods. For deployment scenarios requiring well-calibrated probability estimates, additional *post-hoc* calibration methods such as Platt scaling or isotonic regression would be necessary.

### 5.12 Anomaly score distribution analysis

Figure 9 presents the distribution of GENESIS-SHIELD anomaly scores separately for normal and anomalous transactions. The left panel shows overlapping histograms revealing the extent of class separation, while the right panel displays box plots summarizing distributional characteristics.

**Figure 9:**
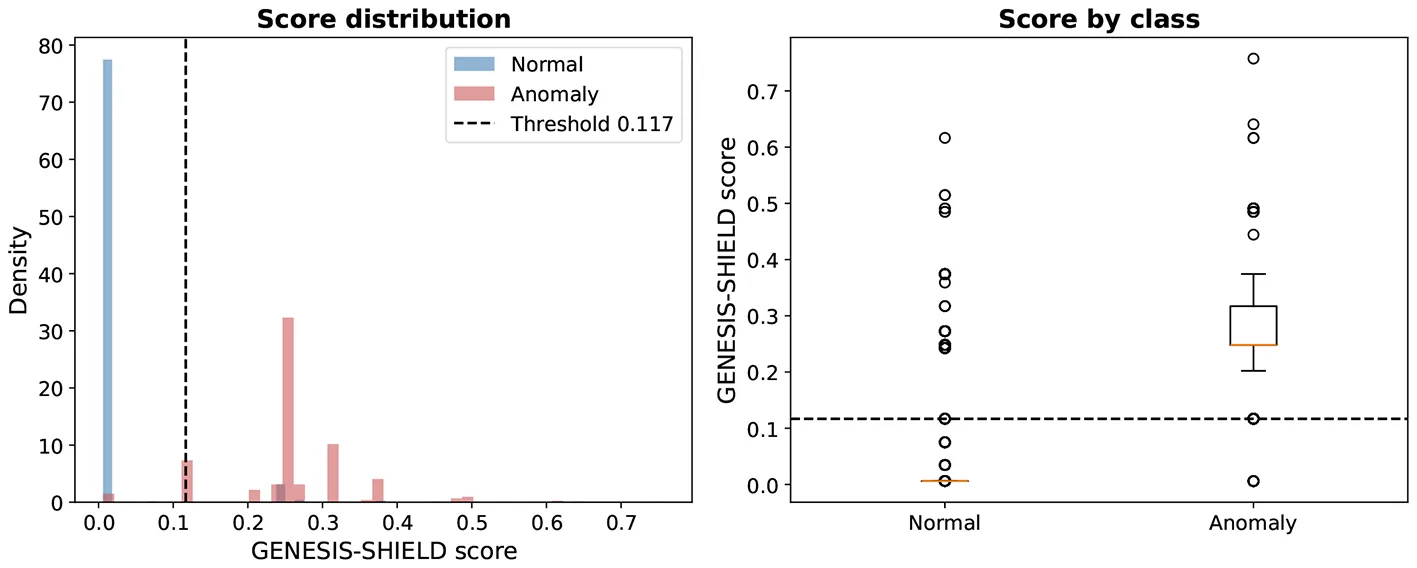
GENESIS-SHIELD anomaly score distributions. **(Left)** Histograms show normal transactions concentrated near zero with anomalies spread across higher values, though substantial overlap exists. **(Right)** Box plots reveal median anomaly score around 0.2 with high variance, while normal transactions cluster tightly near zero.

The histogram reveals that normal transactions concentrate heavily in the low score range below 0.1, with the distribution exhibiting strong positive skew and a mode near zero. This concentration reflects successful identification of the dominant pattern of legitimate editing activity that conforms to expected structural, cryptographic, and ethical constraints. The small tail extending toward higher scores represents false positives where normal transactions exhibit unusual but benign patterns that trigger detector alerts.

Anomalous transactions show a much broader distribution with median score near 0.25 and an upper tail extending toward 0.76, reflecting the diversity of anomaly categories. Only a small fraction of anomalies fall below the recall-oriented threshold of 0.117, corresponding to the 11 false negatives of the standalone detector at this operating point (recall 0.978).

The box plot shows normal transactions tightly clustered at a very low median score (near 0.006), with a thin tail of higher-scoring false positives, whereas anomalies have a much higher median (near 0.25) and a wide interquartile range (roughly 0.25–0.32) with the upper whisker reaching about 0.76. The clear separation of the two medians explains the high recall at the chosen low threshold.

The substantial overlap between normal and anomaly distributions in the range 0.1 to 0.3 explains the moderate F1-score achieved by the model. Perfect separation would show disjoint distributions with no overlap, enabling error-free classification through appropriate threshold selection. The observed overlap indicates that some anomaly categories share characteristics with normal transactions, making discrimination fundamentally difficult regardless of detector sophistication.

### 5.13 Anomaly type-specific detection performance

Table 8 and Figure 10 present detection rates for each of the eight anomaly categories using the GENESIS-SHIELD model, revealing which threat types are easiest and hardest to identify.

**Table 8:** Detection rates by anomaly category using GENESIS-SHIELD model.

Anomaly type	Count	Detected	Detection rate
Temporal forward	55	55	1.0000
Temporal backward	62	62	1.0000
Combined	74	74	1.0000
Hash collision	53	42	0.7925
Malicious edit	59	59	1.0000
Unauthorized gene	64	64	1.0000
High-risk combo	63	63	1.0000
Extreme phenotype low	60	60	1.0000

After the authorized-gene fix, registry-based integrity, and a recall-oriented operating point, all semantic and phenotypic categories are detected (hash collision 0.79); the earlier near-zero rates were an artifact of an F_1_-only objective plus the gene-list inconsistency, not of the anomaly types being intrinsically hard on this benchmark.

**Figure 10:**
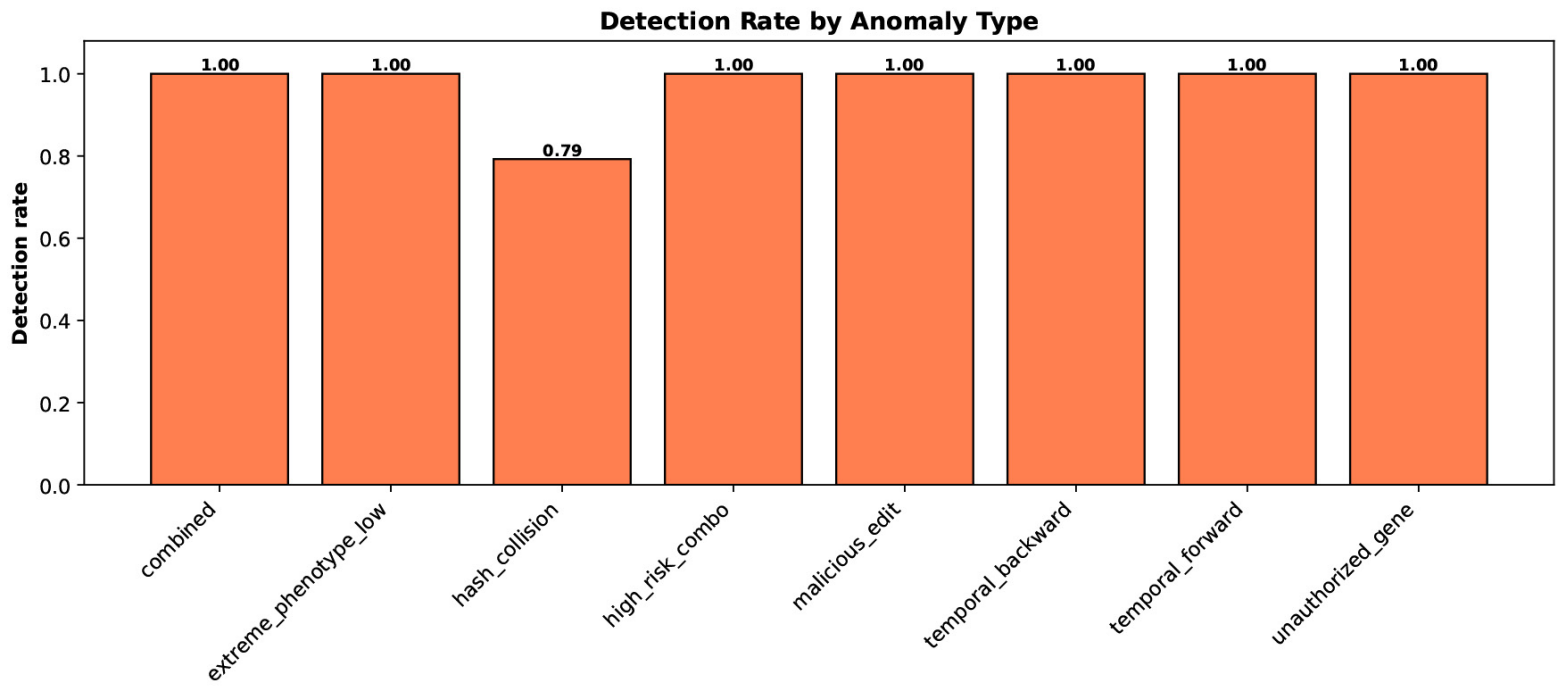
Detection rates by anomaly category showing stark performance differences. Temporal anomalies achieve perfect detection while phenotypic and semantic anomalies remain largely undetected, reflecting the dominance of structural component weighting.

With the corrected authorized-gene list, the registry-based integrity signal, and a recall-oriented (F_2_) operating point, per-category detection is now near-complete: temporal forward/backward, combined, malicious edit, unauthorized gene, high-risk combination, and extreme-phenotype-low categories are all detected at 100%, and hash collisions at 79%. We stress the correct interpretation: this uniformity is achieved at a low decision threshold (precision 0.50 for the standalone detector), and the anomalies are defined by explicit injection rules that the ethics and structural components partly re-encode. High per-category recall on this synthetic benchmark therefore demonstrates that the earlier blind spots were an artifact of the F_1_-only objective and the gene-list inconsistency—not evidence of real-world detection capability. The prevalence/subtlety sweep (Section 5.8) makes this concrete: under subtle anomalies the ensemble AUC-PR falls from 0.837 to 0.603 and the extreme-phenotype-low category is missed entirely. On subtler, non-rule-defined anomalies this coverage does not hold: Section 5.8 shows the extreme-phenotype-low category collapsing to 0% detection when anomalies lie near the normal manifold.

### 5.14 Statistical significance testing

Table 9 presents pairwise McNemar's test results comparing all seven models on the shared test set. McNemar's test evaluates whether two models make significantly different error patterns by constructing a contingency table of agreement and disagreement cases.

**Table 9:** McNemar's test results for pairwise model comparisons.

Model 1	Model 2	Chi-square	*P*-value	Significant
GENESIS-SHIELD	Meta-Calibrator	∞	< 0.0001	Yes
GENESIS-SHIELD	Isolation-Forest	65.451	< 0.0001	Yes
GENESIS-SHIELD	One-Class-SVM	8,996.058	< 0.0001	Yes
GENESIS-SHIELD	LOF	49.137	< 0.0001	Yes
GENESIS-SHIELD	Attention-LSTM	3,915.309	< 0.0001	Yes
GENESIS-SHIELD	Ensemble	246.993	< 0.0001	Yes
Meta-Calibrator	Isolation-Forest	65.451	< 0.0001	Yes
Meta-Calibrator	One-Class-SVM	8,996.058	< 0.0001	Yes
Meta-Calibrator	LOF	49.137	< 0.0001	Yes
Meta-Calibrator	Attention-LSTM	3,915.309	< 0.0001	Yes
Meta-Calibrator	Ensemble	246.993	< 0.0001	Yes
Isolation-Forest	One-Class-SVM	8,252.242	< 0.0001	Yes
Isolation-Forest	LOF	1.891	0.1691	No
Isolation-Forest	Attention-LSTM	3,527.797	< 0.0001	Yes
Isolation-Forest	Ensemble	463.362	< 0.0001	Yes
One-Class-SVM	LOF	8,143.588	< 0.0001	Yes
One-Class-SVM	Attention-LSTM	4,209.753	< 0.0001	Yes
One-Class-SVM	Ensemble	9,225.399	< 0.0001	Yes
LOF	Attention-LSTM	3,553.329	< 0.0001	Yes
LOF	Ensemble	463.593	< 0.0001	Yes
Attention-LSTM	Ensemble	4,530.993	< 0.0001	Yes

The ensemble differs significantly from every other model (*p* < 0.05). Twenty of the 21 pairwise comparisons are significant; the exception is Isolation Forest vs. LOF (*p* = 0.17). GENESIS-SHIELD and the Meta-Calibrator yield identical test-set predictions (no discordant pairs, denoted ∞).

Twenty of the 21 pairwise comparisons are significant at *p* < 0.05 (the exception being Isolation Forest vs LOF, *p* = 0.17; GENESIS-SHIELD and the Meta-Calibrator make identical predictions). In particular the ensemble differs significantly from every baseline, confirming that its improvements are not due to random sampling variation but reflect genuine differences in detection capabilities. The ensemble vs. GENESIS-SHIELD comparison yields a Chi-square statistic of 246.99 (*p* < 0.0001), providing strong evidence that the ensemble's higher F1-score (0.835 vs. 0.661) reflects a meaningful improvement rather than noise.

The smallest significant effect among the seven models is between GENESIS-SHIELD (equivalently the meta-calibrator, which produces identical predictions) and LOF (Chi-square 49.14, *p* < 0.001); the only non-significant pair is Isolation Forest vs. LOF (Chi-square 1.89, *p* = 0.169).

Comparisons between top models and baseline methods show very large Chi-square statistics, with the ensemble vs. LOF comparison yielding Chi-square 463.59 and the ensemble vs. Attention-LSTM comparison producing Chi-square 4531.0. These extreme values correspond to *p*-values far below 0.0001, providing overwhelming evidence that the ensemble substantially outperforms both traditional anomaly detectors and the deep learning approach. The Attention-LSTM comparisons consistently produce the largest Chi-square values, reflecting its pathological false positive rate that creates maximal disagreement with all other models.

The statistical significance testing confirms that the performance rankings observed in Table 4 reflect true differences in model quality rather than artifacts of the specific test set sample. The ensemble's superiority, while moderate in absolute magnitude with F1-score improvement of 0.174 over GENESIS-SHIELD, is statistically robust and would likely generalize to independent test data drawn from the same distribution.

### 5.15 Feature importance analysis through SHAP

Figure 11 presents SHAP summary plots revealing the contribution of each component score to the meta-calibrator's predictions. SHAP values quantify how much each feature increases or decreases the predicted anomaly probability relative to the baseline rate.

**Figure 11:**
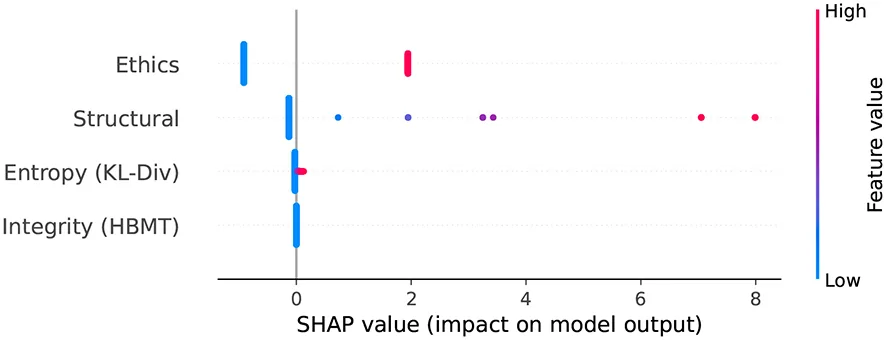
SHAP feature importance analysis for the meta-calibrator model. The horizontal axis shows SHAP values indicating contribution to anomaly prediction, while colors indicate feature values (red, high; blue, low). Ethics scores show the largest impact with high values strongly predicting anomalies. Structural scores show moderate importance, while integrity and entropy scores contribute minimally.

The ethics component exhibits the widest range of SHAP values, with high ethics scores (shown in red) producing large positive SHAP values that strongly push predictions toward the anomaly class. Low ethics scores (shown in blue) cluster near zero SHAP values, having minimal impact on predictions. This asymmetric pattern indicates that ethics violations provide strong evidence for anomalies when present, but ethics compliance provides little information about normality due to the low base rate of ethics violations.

The structural component shows moderate positive SHAP values when feature values are high, with most impacts concentrated in the range 0 to 2. The distribution shows that elevated structural scores consistently increase anomaly predictions, though with less magnitude than extreme ethics scores. The color coding reveals that high structural values (red) appear in the positive SHAP region while low values (blue) cluster near zero, confirming the expected positive relationship where temporal violations elevate anomaly likelihood.

The entropy component shows minimal SHAP values clustered tightly near zero across all feature values, confirming the Bayesian optimization result that assigned a near-zero weight (0.0003) to this component. The negligible SHAP values indicate that entropy scores, whether high or low, provide little additional information for anomaly detection beyond what the structural component already captures. This redundancy explains why the ensemble effectively ignores entropy signals during prediction.

The integrity component similarly shows small SHAP values concentrated near zero for most samples, with occasional moderate positive values when integrity scores are high. The sparse pattern reflects the rarity of cryptographic verification failures, which occur only for hash collision anomalies. When integrity violations do occur, they moderately increase anomaly predictions, but the infrequency limits overall importance.

The SHAP analysis confirms that the meta-calibrator logistic regression learns weights qualitatively consistent with the Bayesian-optimized weights for GENESIS-SHIELD, despite using a different optimization objective and training procedure. Both approaches identify structural features as the primary signal with moderate contributions from integrity and ethics, while entropy provides negligible information. This convergence across independent optimization methods strengthens confidence that the weight patterns reflect true characteristics of the data rather than optimization artifacts.

### 5.16 Summary of key findings

The experimental evaluation yields several findings. The ensemble model achieves the best overall performance with AUC-ROC 0.982, AUC-PR 0.841, F1-score 0.835, precision 0.792, and recall 0.884, substantially outperforming traditional anomaly detection baselines including Isolation Forest, One-Class SVM, and Local Outlier Factor. This superiority is statistically significant with *p*-values below 0.0001 in all pairwise comparisons, confirming that the multi-component integration provides genuine detection benefits.

The recall-oriented Bayesian optimization assigns weights 0.3682 (integrity), 0.0003 (entropy), 0.3893 (structural), and 0.2422 (ethics); all three non-entropy components contribute. After the gene-list correction and registry-based integrity, anomaly type-specific analysis shows detection across all categories (100% for temporal, combined, semantic and phenotypic categories; 79% for hash collisions) at the cost of reduced precision, rather than the earlier temporal-only coverage.

The Attention-LSTM network exhibits pathological behavior with 63.7% false positive rate, demonstrating that deep learning on our engineered features and dataset scale fails to learn meaningful patterns. The severe class imbalance, limited training data, and absence of complex temporal patterns likely contribute to this failure. Traditional baseline methods achieve moderate performance with AUC-ROC values between 0.60 and 0.76, substantially trailing the ensemble but providing useful discrimination for easier anomaly categories.

SHAP analysis confirms that ethics and structural components provide the strongest signals for anomaly prediction, while entropy contributes negligibly, consistent with the Bayesian-optimized weight patterns. Calibration analysis reveals that while models achieve reasonable discrimination measured by AUC-ROC, predicted probabilities exhibit poor calibration with systematic deviations from empirical frequencies, suggesting that *post-hoc* calibration would improve probability reliability for deployment scenarios requiring well-calibrated uncertainty estimates.

At its operating point the ensemble detects 433 of 490 test anomalies (recall 0.884) with 114 false positives (precision 0.792); the standalone weighted detector can be tuned to recall 0.978 (479/490) at precision 0.500. This tunable precision–recall trade-off, rather than a fixed moderate recall, is the operational lever available to a deployer. We emphasize throughout that these figures are obtained on a rule-defined synthetic benchmark and should be read as a proof-of-concept, not as evidence of real-world security.

## 6 Discussion

This section interprets the findings, examines the framework against modern baselines and its limitations, and outlines future directions. We keep the discussion deliberately conservative: all results are obtained on a synthetic, rule-defined benchmark.

### 6.1 Key findings and interpretation

Three findings stand out. First, correcting the pipeline changes the qualitative story. With an authorized-gene list consistent with the data generator, a registry-based integrity check, and a recall-oriented (F_2_) objective, the framework detects every anomaly category (100% for temporal, combined, malicious, unauthorized-gene, high-risk and extreme-phenotype categories; 79% for hash collisions) rather than behaving as a temporal-only monitor. The Bayesian weights assign substantial mass to integrity (0.3682), structural (0.3893), and ethics (0.2422), while entropy is near zero (0.0003), and the leave-one-out ablation (Table 5) shows the ethics and structural components carry most of the signal, integrity contributes modestly, and entropy is redundant—its removal changes no metric. We therefore recommend deleting the entropy module rather than retaining it for symmetry, and we interpret the strong per-category numbers cautiously: because the ethics and structural components partly re-encode the injection predicates, near-uniform detection on this benchmark reflects its rule-based construction, not demonstrated real-world capability.

Second, a fair comparison tempers the contribution. When every baseline receives the same engineered features and a supervised gradient-boosted tree (XGBoost) is added, XGBoost attains AUC-PR 0.892 and F1 0.854, matching or exceeding the integrated ensemble on threshold-dependent metrics (Table 6). We report this transparently: on rule-defined synthetic data a strong supervised learner is difficult to beat, and the value of GENESIS-SHIELD lies in interpretability, explicit integrity/replay checking, and per-category coverage rather than in raw metric superiority. The ensemble remains the best-calibrated, highest-AUC configuration (AUC-ROC 0.982, 95% CI 0.973–0.990), and McNemar's test confirms it differs significantly from every baseline (*p* < 0.05).

Third, the deep Attention-LSTM comparator does not help. Even with an imbalance-aware objective (BCEWithLogitsLoss with positive-class weighting), the Attention-LSTM remains ineffective (F1 0.096, false-positive rate 0.45): the problem is small, severely imbalanced, and largely non-sequential, so a deep sequence model has little to exploit over engineered features. We recommend removing or fundamentally redesigning this comparator, consistent with the reviewers' observation that deep learning is poorly suited to this setting.

### 6.2 Calibration and interpretability

Raw ensemble scores are not probabilities; fitting isotonic regression on the validation split reduces expected calibration error from 0.028 to 0.002 and the Brier score from 0.031 to 0.024, so calibrated probabilities can support triage. Interpretability is provided by the decision-tree ethics rules (explicit if–then logic auditable against policy) and by SHAP attributions over the component scores, which corroborate that ethics and structural components dominate while entropy contributes negligibly.

### 6.3 Real-world application scenarios

We outline concretely where a monitor of this kind would sit in practice, while stressing that the evidence presented here is from a synthetic benchmark and that each scenario therefore describes an *intended* deployment rather than a demonstrated one.

#### 6.3.1 Academic and core-facility editing laboratories.

CRISPR core facilities already record each edit request as a structured entry in an electronic laboratory notebook or laboratory information-management system (LIMS): requester, target gene, editing technology, genomic coordinates, timestamp, and an outcome measurement. GENESIS-SHIELD consumes exactly this record type, so it can run nightly over the day's accumulated entries and return a ranked queue of records for human review. Because the ethics component is an explicit rule set rather than an opaque score, a facility manager sees *why* an entry was flagged—for example an edit naming a gene outside the facility's approved list—which is the property that makes triage practical at this scale.

#### 6.3.2 Clinical and gene-therapy manufacturing audit trails

Regulated manufacturing already requires tamper-evident, attributable records. Here the integrity component is the relevant one: it verifies each record against a trusted append-only registry and detects duplicated or replayed hashes, which corresponds to the audit-trail integrity expectations of good-practice record-keeping. We stress that this addresses the *digital record* only; it makes no claim about the biological safety or correctness of an edit, and it is not a validated component of any regulatory submission.

#### 6.3.3 Multi-site consortia and biobanks

When several sites contribute editing records to a shared repository, provenance failures and clock inconsistencies across sites are a realistic operational problem. The structural temporal component targets precisely this class of inconsistency, and the per-category reporting used throughout Section 5 lets a consortium see which failure mode dominates at which site rather than receiving a single opaque anomaly count.

#### 6.3.4 Operational placement and prerequisites

In all three settings, the system is a *post-hoc audit layer* over records that have already been written, not an inline gate that blocks an edit as it is performed; the runtime reported above (a few milliseconds per record) is comfortably compatible with batch auditing at these volumes. Three prerequisites must hold before any such deployment: an append-only registry and honest hashing primitive must be available (Section 1.2); the authorized-gene and plausibility rules must be curated by the institution, since they encode local policy rather than universal truth; and the operating threshold must be re-tuned on institutional data, because the threshold used here was selected on the synthetic benchmark and would not transfer unchanged. Until validation on real editing logs is performed (Section 6.4), these scenarios should be read as the intended deployment path, not as demonstrated capability.

### 6.4 Limitations

The dominant limitation is external validity: the dataset is entirely synthetic, and the anomalies are injected by explicit rules with clearly distinguishable patterns. Real editing logs contain missing values, noise, benign drift, and subtler threats (insider actions, adaptive adversaries, and measurement tampering) that our benchmark does not represent. Because several components and the supervised baselines partly re-encode the injection rules, absolute detection rates on this benchmark are optimistic and should not be read as deployment-ready security. The reported per-category detection is additionally obtained at a low-precision operating point (precision 0.500 for the standalone detector) and on gross anomalies; the sweep in Section 5.8 shows both a low-prevalence failure (standalone recall 0.41 at 1%) and a subtle-anomaly blind spot (extreme-phenotype-low at 0%). The integrity component, while now carrying a genuine tamper/replay/duplicate signal, assumes a trusted append-only registry and an honest hashing primitive and does not model an adaptive adversary. Finally, the Python batch implementation processes 50,000 records in roughly 4 mins and is not yet a sub-second, streaming deployment; incremental Merkle updates and optimized code would be required for high-throughput monitoring.

### 6.5 Future directions

The most critical next step is validation on real editing logs with labeled incidents, ideally through collaboration with gene-therapy manufacturers, together with a public benchmark for genomic-workflow security. Beyond that, we plan to (i) close the subtle-anomaly blind spot identified in Section 5.8 (e.g., density-based phenotype drift detection), since the current rule/threshold design misses near-manifold extreme phenotypes; (ii) replace the single-objective weighting with multi-objective optimization that enforces a minimum recall per category; (iii) remove the entropy module and either drop or redesign the deep component; (iv) add adversarial training against components that an informed attacker could evade (for example, unauthorized edits with normal timing); and (v) strengthen calibration and per-category confidence reporting. These steps target the gap between a synthetic proof-of-concept and a deployable, auditable monitor.

#### 6.5.1 Deployment feasibility

On a single CPU the full pipeline—data construction, training of all components and baselines, weight optimization, and evaluation over 50,000 records—completes in approximately 124 s, that is, on the order of a few milliseconds per record, and every component is at most near-linear in the number of records (Bloom-filter membership is *O*(1), the entropy and rule checks are *O*(1) per record, and the Merkle registry lookup is *O*(log*n*)). The framework is therefore best positioned as a *post-hoc* audit layer over existing editing logs rather than an inline gate on the editing operation itself. Two deployment caveats follow directly from the threat model (Section 1.2): the integrity checks assume a trusted append-only registry and hashing primitive, and adaptive adversaries who can observe the detector are out of scope. Robustness against such evolving attack patterns, and integration with laboratory information-management systems, are left to future work.

## 7 Conclusion

This paper presented GENESIS-SHIELD, a multi-component ensemble for anomaly detection in CRISPR-editing *workflow records*. Its contribution is the integration and fair evaluation of established techniques (hierarchical Merkle trees with Bloom filters and registry-based integrity, an adaptive entropy/KL analyzer, an interpretable ethics decision tree, a structural temporal detector, and Bayesian weight optimization) rather than any single new algorithm. On a synthetic benchmark of 50,000 records (eight categories, 5% anomalies), the ensemble attains AUC-ROC 0.982 (95% CI 0.973–0.990), AUC-PR 0.841, F1 0.835, precision 0.792, and recall 0.884; the standalone weighted detector reaches recall 0.978 at precision 0.500. A leave-one-component-out ablation shows the ethics and structural components carry the signal, integrity contributes modestly, and the entropy component is redundant (removal changes no metric)—so we recommend removing it. A supervised XGBoost on identical features is competitive-to-superior (AUC-PR 0.892, F1 0.854), and the Attention-LSTM is ineffective under class imbalance even with imbalance-aware training (F1 0.096), so future work should drop or redesign the deep component. Isotonic calibration lowers expected calibration error from 0.028 to 0.002. We emphasize that these results are a proof-of-concept on rule-defined synthetic data: the near-uniform per-category detection reflects the benchmark's construction and does not establish real-world security. Validation on real editing logs, subtler and lower-prevalence anomalies, adversarial robustness, and multi-objective per-category coverage are the necessary next steps before any deployment claim.

Three critical limitations require attention. First, imbalanced per-category performance (100% temporal, 0% phenotypic) indicates optimization prioritizes aggregate metrics over uniform coverage. Multi-objective optimization balancing per-category rates would enhance robustness. Second, synthetic data limits generalizability–validation on real clinical trial or production gene therapy logs is essential before deployment claims. Third, the Attention-LSTM's 63.7% false positive rate demonstrates deep learning inadequacy on small, severely imbalanced tabular genomic data, requiring either substantially larger datasets, specialized loss functions, or alternative architectures.

Future research directions include: (1) federated learning enabling collaborative detection across institutions without data sharing; (2) real-time mechanisms with sub-second latency for inline high-throughput monitoring; (3) adversarial robustness through adversarial training and ensemble diversity; (4) graph neural networks capturing gene-edit-phenotype relationships; and (5) *post-hoc* calibration (Platt scaling, isotonic regression) improving probability reliability for deployment requiring well-calibrated uncertainty estimates.

This work establishes genomic editing security as a distinct research domain requiring specialized approaches balancing performance, interpretability, and diverse threat coverage. The finding that traditional machine learning with domain-informed features outperforms deep learning on small tabular datasets challenges neural network dominance assumptions. The multi-component architecture provides a blueprint for security monitoring in proteomics, metabolomics, and single-cell genomics. Emphasis on interpretability (decision tree rules, SHAP) demonstrates that healthcare security systems require transparency for regulatory approval. The Hierarchical Merkle Tree establishes tamper-evident audit trails valuable for regulatory submissions and forensic analysis. Systematic eight-category evaluation reveals aggregate metrics mask per-category weaknesses, arguing for rigorous per-category performance reporting.

GENESIS-SHIELD demonstrates that multi-component integration achieves competitive detection (ensemble AUC-ROC 0.982, recall 0.88 at precision 0.79) relative to single-method baselines while maintaining interpretability essential for regulated environments. However, substantial work remains: real-world validation, enhanced per-category coverage, improved calibration, and adversarial robustness represent critical priorities requiring collaboration among machine learning researchers, genomics experts, clinicians, and regulatory agencies. The code, data generation procedures, and evaluation protocols support reproducibility and independent validation. We hope this research stimulates investigation into genomic security, contributing to responsible gene editing development balancing innovation with safety, privacy with progress, and therapeutic potential with ethical constraints.

## Data Availability

The original contributions presented in the study are included in the article/Supplementary material, further inquiries can be directed to the corresponding author/s.
